# Mechanistic insight to ROS and Apoptosis regulated cytotoxicity inferred by Green synthesized CuO nanoparticles from *Calotropis gigantea* to Embryonic Zebrafish

**DOI:** 10.1038/s41598-017-16581-1

**Published:** 2017-11-24

**Authors:** Puja Kumari, Pritam Kumar Panda, Ealisha Jha, Khushboo Kumari, Kumari Nisha, M. Anwar Mallick, Suresh K. Verma

**Affiliations:** 1 Advance Science & Technology Research Centre, Vinoba Bhave University, Hazaribagh, Jharkhand 825301 India; 2 Division of Paediatric Haematology and Oncology, University Children’s Hospital, University of Freiburg, Freiburg, 79106 Germany; 3 Memorial University of Newfoundland, Department of Physics and Physical Oceanography, St. John’s, Newfoundland and Labrador, Labrador, NL A1C 5S7 Canada; 4 School of Biotechnology, KIIT University, Bhubaneswar, Odisha 751024 India

## Abstract

With the rapid development of nanotechnology, much has been anticipated with copper oxide nanoparticles (CuO NP) due to their extensive industrial and commercial application. However, it has raised concern over the environmental safety and human health effects. In this study, CuO nanoparticles were synthesized using the green method with floral extract of *Calotropis gigantea* and characterized by standard physiochemical techniques like DLS, Zeta potential determination, UV- Visible Spectroscopy, XRD, FTIR, FESEM, and TEM. Mechanistic cytotoxicity studies were performed using experimental and computational assays including morphological analysis, hatching, and viability rate analysis along with ROS and apoptosis analysis. Physiochemical characterization of CuO NP determined the size and zeta potential of synthesized nanoparticles to be 30 ± 09 nm to 40 ± 2 nm and −38 mV ± 12 mV respectively. Cytotoxicity evaluation with Zebrafish revealed malfunctioned organ development with differential viability and hatching rate at 48 hpf and 72 hpf with LC50 of 175 ± 10 mg/l. Computational analysis depicted the influential role of CuO nanoparticles on zebrafish embryo’s he1a, sod1 and p53 functional expression through hydrophobic and hydrogen bond interaction with amino acid residues. Study demonstrated valuable information of cytotoxic impact which can be influential in further studies of their eco-toxicological effects.

## Introduction

Copper oxide nanoparticles have long been used as an antimicrobial agent in medical, cosmetics industry and appliances for public use^1^. The increased demands with this utility have instigated a large production and use of these nanoparticles day by day. This bulk production and usage have raised the concern over the toxicity of these nanomaterials on the ecosystem as well as on human health. Concerns over the toxic effect of CuO nanoparticles usage has drawn a specific attraction of toxicology researchers in last few years. Many studies have been reported the cytotoxicity and genotoxicity of CuO nanoparticles through *in vitro* and *in vivo* investigation in mammalian cell line and animal models. *In vitro* studies have reported the cytotoxicity and genotoxicity of CuO nanoparticles in lung epithelial, peripheral blood and cancer cell lines^2–5^. *In vivo* studies have also revealed the genotoxic effects of neoplastic lesions, DNA alteration and DNA strand break in mouse^6,7^. Used CuO nanoparticles released in the aquatic environment also cause toxicity to fishes and aquatic animals. So, synthesis of eco-friendly biocompatible CuO nanoparticles has become the urgent need of the hour with a proper investigation of their biological properties like cytotoxicity.

A number of previous literatures have reported the synthesis of CuO nanoparticles using a variety of methods. Some of them includes chemical synthesis^8^, physical synthesis^9^ and biological synthesis^10^. Physical synthesis has been reported to have the issue of contamination however chemical synthesis is known for use of chemicals which could be harmful to the ecosystem as well as human health^11^. Using biological agents like plants and microbes for synthesis process could be a potential solution to these problems. Moreover, nanoparticles synthesized from biological processes are potent for biomedical applications^11^. Henceforth the synthesis process is commonly called as “Green synthesis”.

In recent years, the Zebrafish (*Danio rerio*) has gained a lot of popularity among scientific communities for the investigation of nanotoxicity of different nanomaterials. The peculiar feature of the short life cycle, transparency of embryos and larvae, low-cost easy maintenance in lab scale of this organism make it a suitable *in vivo* model for all types of research. Many nanotoxicologists have evaluated the nanotoxicity of different nanomaterials like Zinc oxide nanoparticles, Titanium oxide nanoparticles in Zebrafish model^12,13^. Sun *et al*.^5^ and Ganeshan *et al*.^14^ have recently reported the effect of commercially available CuO nanoparticles on developing Zebrafish and the induction of oxidative stress and teratogenicity in them. Moreover, toxicological studies in Zebrafish model have an advantage of mimicking to human model because of their genetic similarities with humans^15^.

Although studies have been reported on the toxicity of CuO nanoparticles with Zebrafish, the knowledge about the investigation of the green synthesized CuO nanoparticles are limited. With this study, we are reporting a novel green synthesis of CuO nanoparticles with the help of aqueous floral extract of *Calotropis gigantea*. Moreover, investigation of *in vivo* nanotoxicity in Zebrafish model has also been reported at the cellular level by experimental and computational analysis. Specifically, we have synthesized CuO nanoparticles by a green method to achieve less cytotoxicity than the commercially available CuO nanoparticles and characterized their physiochemical properties. We also determined their toxicological behavior in Zebrafish by analyzing their effect on developmental and morphological changes with detail assessment of induced oxidative stress and cellular apoptosis. These studies will provide a new direction for the synthesis of biocompatible CuO nanoparticles and might help the future effective nanotoxicological investigation on their exposure.

## Materials and Methods

### Green Synthesis of CuO nanoparticles (CuO NP)

**Figure 1:**
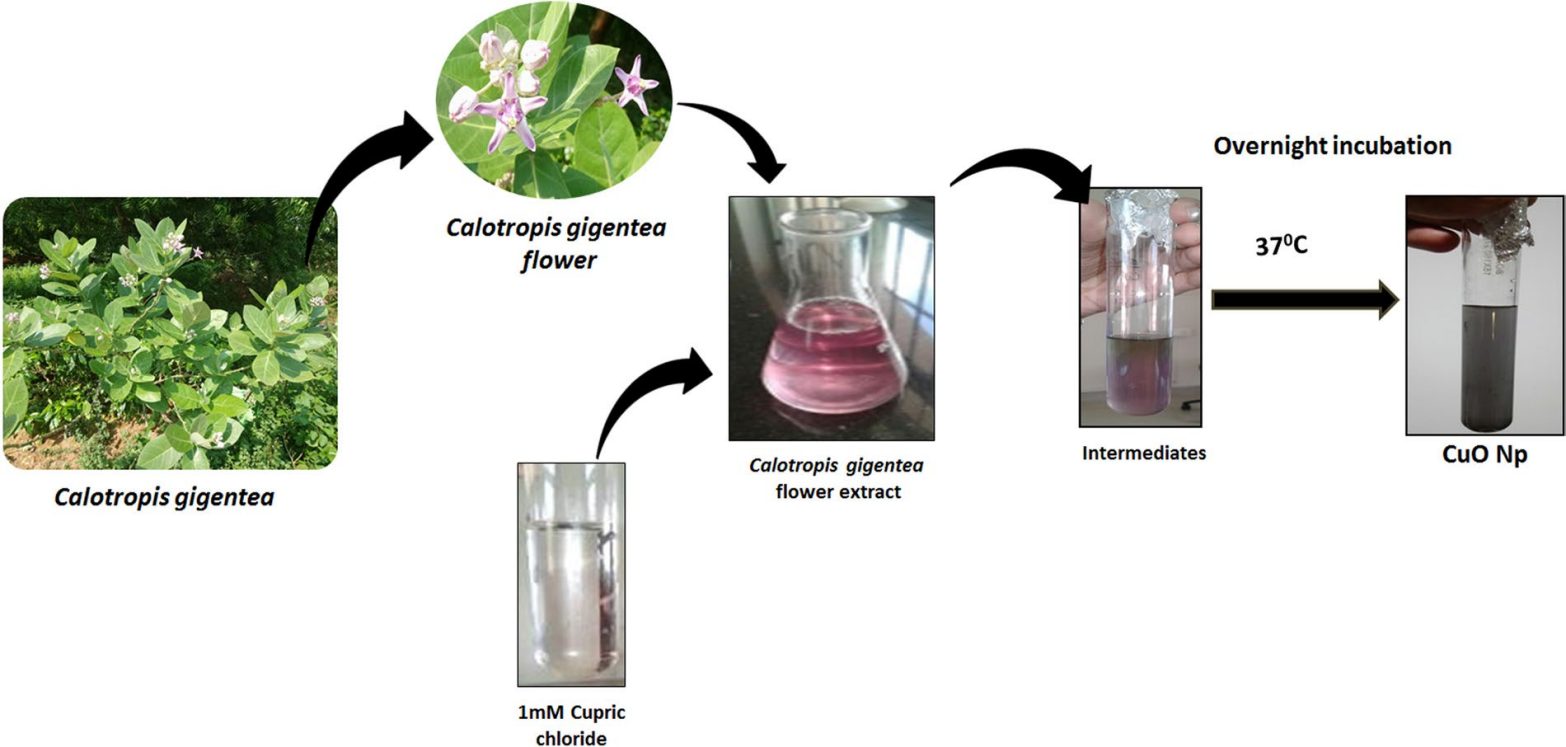
Schematic diagram of green synthesis of CuO nanoparticles from floral extract of *Calotropis gigantea*.

Green synthesis of CuO nanoparticle (CuO NP) was done by the reduction and stabilization of Copper chloride salt (CuCl_2_) with the help of floral extract of *Calotropis gigantea* extracted in an aqueous medium. Flowers of *Calotropis gigantea* were collected from botanical garden of Department of Botany, Vinoba Bhave University at morning 10 am. 10 g floral parts of flowers were chopped off minutely and boiled in 100 ml of distilled water for 15 min till the appearance of pink color. After boiling, the solution was cooled and sieved with the help of muslin cloth to separate the extract. The synthesis reaction was set up by incubating floral extract with aq. 1 mM CuCl_2_ solution at 37 °C for overnight (Fig. 1). After incubation, the synthesized CuO nanoparticles were centrifuged at 10000 rpm for 5 min and washed two times with distilled water to get rid of unused biomolecules in solution. The centrifuged precipitate of CuO NP was dried and diluted with distilled water for further physiochemical characterization. All data generated or analyzed during this study are included in this published article.

### Identification of bioactive compounds of *Calotropis gigantea* floral extract

Identification of bioactive compounds responsible for the synthesis of CuO NP which was present in the floral extract of *Calotropis gigantea* was done with the help of GC-MS analysis. The floral extract obtained before and after synthesis was dried and dissolved in methanol for analysis of used bioactive compound in nanoparticle formation. An Agilent gas chromatograph model 6890 N coupled to an Agilent 5973 N mass selective detector was used for GC-MS analysis. Separation of analysts was done on an HP-5MS capillary column (30 m × 0.25 mm × 1.0 μl) by applying 40–70 °C for 10 min. Transfer line temperature was 280 °C. Conditions for mass detector were: electronic impact (EI) mode at 70 eV; source temperature: 230 °C; scanning rate 2.88 scan S-1; mass scanning range: m/z 29–540. The carrier gas was helium at 1.0 ml min-1. The tentative volatile components were identified by comparing the mass spectra with the data system library (NIST 98) supported by retention index data.

### Physiochemical characterization of CuO NP

Green Synthesized CuO NP was characterized for their physiochemical properties with the help of different physical techniques like UV-Vis spectroscopy, Dynamic light scattering, X-ray diffraction, Fourier transform infrared spectroscopy and Field emission Scanning electron microscopy (FESEM). The size of CuO NP was determined and validated by FESEM (Carl Zeiss) equipped with EDS (Oxford, Inca) and TEM. For FESEM analysis, Aq. solutions of CuO NP were dried in hot sun and imaging was performed at 20KV. The size and stability of CuO NP in aqueous medium were also evaluated by determination of hydrodynamic diameter and zeta potential by Zetasizer (Malvern, UK) in Holtfreter medium^16^ after making fresh suspension and after 72 h. Optical properties of fresh suspension CuO NP and after 72 h were analyzed by visualization of SPR peak obtained from UV-Vis spectra scan from 200 nm–800 nm in UV-Visible spectrophotometer (Cary 5000, Agilent, USA). X-ray diffractometer (X-PERT- PRO, Pan Analytical) with CuKα radiation (λ = 0.15418 nm) over a wide range of angle of 20° to 80°was used for structural analysis by XRD techniques. For Fourier transform infrared (FTIR) spectroscopy, Perkin Elmer RXI FTIR spectrometer with an ATR attachment was used to carry out measurements in the range of 400–4000 cm^−1^ at a resolution of 4 cm^−1^.

### Zebrafish and embryo maintenance

All animal procedures were approved by the relevant guidelines of Institutional Animal Ethics Committee (IAEC) of VBU University. All experiments were performed in accordance with relevant animal practice guidelines and regulations of IAEC, VBU University. Zebrafish were obtained from local aquarium fish dealer, Hazaribagh and were maintained in an aquarium setup. The setup was equilibrated with fish water (75 g NaHCO_3_, 18 g sea salt, 8.4 g CaSO_4_ per 1000 ml). Breeding of fishes was induced by keeping males and females in a 3:1 ratio. The photoperiodism was maintained by keeping 14-hours of light and 10 hours of darkness. The water temperature was maintained at 26 ± 2 °C. Prior to breeding, they were fed with an enriched diet along with Bloodworms. Eggs were collected early in the morning and rinsed several times and were reared further in Holtfreter (HF) medium.

### *In vivo* cytotoxicity study in Zebrafish embryo and larvae model

All experiments were performed in accordance with relevant animal practice guidelines and regulations of IAEC, VBU University*. In vivo* cytotoxicity investigations of green synthesized CuO NP in Zebrafish embryo and larvae was done with the standard protocol determined by Zhu *et al*.^17^. In brief, 20 zebrafish embryo of 24 hour post fertilization (hpf) were exposed to CuO NP at a range of concentration of 5 mg/l to 500 mg/l in HF medium for 72 h. The experimental setup was incubated at 28 ± 1 °C at a photoperiod of 14/10 h light/dark. Microscopic observation was done at each interval to visualize the morphological and developmental changes. Hatching rate was determined as a number of embryos hatched by 72 hpf as compared to untreated group. Mortality rate was expressed as a number of dead embryos after 72 hpf as compared to untreated group. In order to determine the biocompatibility of green synthesized CuO nanoparticles compared to commercially available CuO nanoparticles, all the assays were also conducted in presence of commercially purchase CuO nanoparticles (Merck). All the experiments were repeated thrice. Images were taken in inverted bright field microscope (EVOS, ThermoScientific).

### Oxidative stress analysis in Zebrafish embryos exposed to CuO NP

Induction of oxidative stress in Zebrafish embryos was analyzed by assessment of Reactive Oxygen species (ROS) induced by exposure of CuO NP. The assessment was done by flow cytometry with the help of ROS marker H_2_DCFDA fluorescent dye. Zebrafish embryos exposed to green synthesized CuO NP and commercially purchased CuO NP for 72 h were sacrificed by exposing in the chilled buffer. Embryos were further sonicated to prepare a single cell suspension. The single cell suspension was treated with 1.25 mg/l H_2_DCFDA dye for 20 min in dark and then washed with chilled HF buffer. The stained cell suspension was then analyzed by flow cytometry using Attune NextGen flow cytometer (ThermoScientific, USA). To check the interference of CuO NP with H_2_DCFDA dye, incubation of 50 mg/l and 250 mg/l CuO NP suspension in HF medium was done and analyzed by flow cytometry. Data were expressed in the histogram. FSC and SSC dot plot was used to gate out the debris present in the suspension.

### Apoptosis analysis by Acridine orange staining

Analysis of apoptosis in Zebrafish embryos was done with the help of Acridine orange staining (AO).Untreated and treated zebrafish embryos were washed two times with HF buffer after 72 h treatment and exposed to 5 µg/ml AO dissolved in HF for 20 min. Embryos were washed with HF buffer twice after staining to remove extra stains and images were taken in green channel of EVOS inverted fluorescent microscope (ThermoScientific, USA) to compare the apoptosis occurred in Zebrafish embryos due to exposure of synthesized and commercially purchased CuO NP at different concentration.

### Apoptosis analysis by Annexin V-FITC/ PI staining

Quantitative analysis of apoptosis in Zebrafish embryos exposed to green synthesized and commercially purchased CuO NP was performed with the help of Apoptosis kit provided by ThermoScientific, USA. The kit detects the externalization of phosphatidylserine in apoptotic cells using recombinant annexin V conjugated to green-fluorescent FITC dye and dead cells using propidium iodide (PI). The protocols were performed with according to kit instructions.

### *In silico* molecular docking for CuO NP interaction

Molecular docking analysis was done by using Autodock 4.2^18^ with CuO as ligand and he1a, sod1, tp53 as receptor proteins. Chimera^19^ was used for drawing the structure of CuO and its geometry was optimized using Gaussian 03 program. Energy minimization was carried out using Chimera program for the receptor proteins. The parameters for CuO have been set for Autodock 4.2. Grid dimensions were set to 40 × 40 × 40, with a spacing of 1 Å for all the protein receptors. Lamarckian genetic algorithms (LGA) were used for grid dimensions. The population size of 150 with a maximum number of evaluations set to 2500000 and maximal generations were used with the help of Genetic algorithm for docking runs. The post-docking analysis was performed using conformational clustering’s visualized by Chimera and Discovery Studio Visualizer. 2D interaction plots have been derived from the receptor complexes having TiO2 as a ligand by using LigPlot+^20^.

### Statistical analyses

Statistical analyses were performed using GraphPad Prism v6.01 (San Diego, California). Data were analyzed by one way ANOVA followed by t-test with significance set at P < 0.05. Differences between groups were analyzed by Friedman test for sample comparisons.

## Results

### Green synthesis of CuO NP

**Figure 2:**
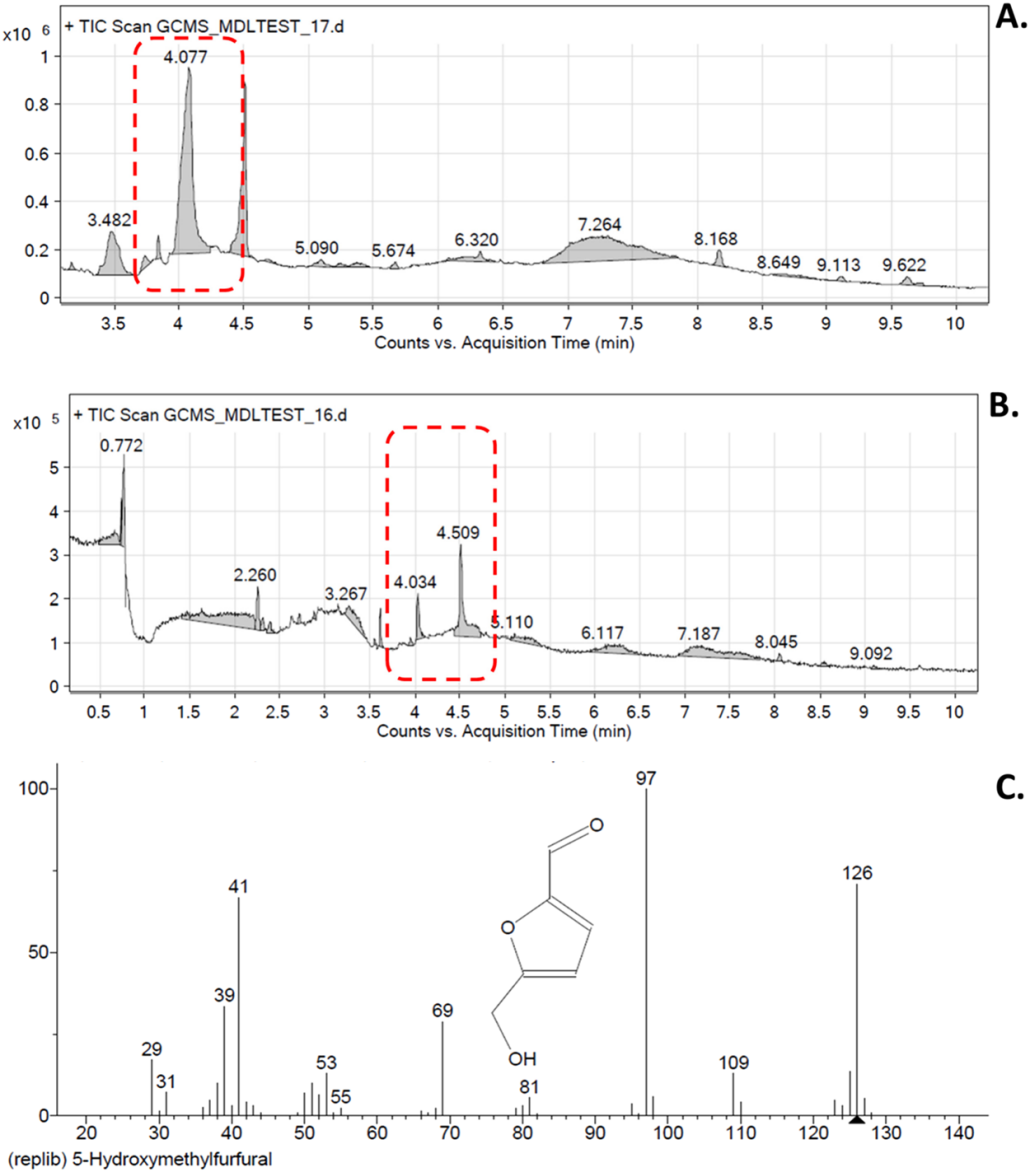
GCMS analyses of floral extract of *Calotropis gigantea*. (**A**) Chromatogram of floral extract before CuO NP synthesis. (**B**) Chromatogram of floral extract after CuO NP synthesis. (**C**) Structural analysis of identified compound at RT 4.077.

Green synthesis of CuO NP was carried out with the help of floral extract of medicinal plant *Calotropis gigantea* as shown in Fig. 1. Faint greenish color with white precipitate was observed after a small incubation time which was further turning into black suspension with black precipitate indicated the synthesis of CuO NP. Probable bioactive present in the floral extract of *Calotropis gigantea* was further investigated by GCMS chromatogram as shown in Fig. 2. The chromatogram of floral extract as shown in Fig. 2A displayed 10 different peaks at different retention time. As indicated in Table 1, Probable identification of compounds corresponding to these peaks revealed the presence of compounds like Folic acid, 5-hydroxymethyl furfural bearing –OH group and saccharides like 6-acetyl b-D-mannose. While comparing the chromatogram of extract used before synthesis (Fig. 2A) and after synthesis (Fig. 2B), it was found that peak corresponding to 5-hydroxymethyl furfural (Fig. 2C) was shifted indicating towards the utilization of the compound during synthesis of CuO nanoparticles.

**Table 1:** Identified compounds at different retention time (RT) peaks determined by GCMS analysis of floral extract of *Calotropis gigantea*.

Retention Time	Identified Compound	Structure
3.482		
4.077		
5.090		
5.674		
6.320		
7.264		

### Size determination of CuO NP

**Figure 3:**
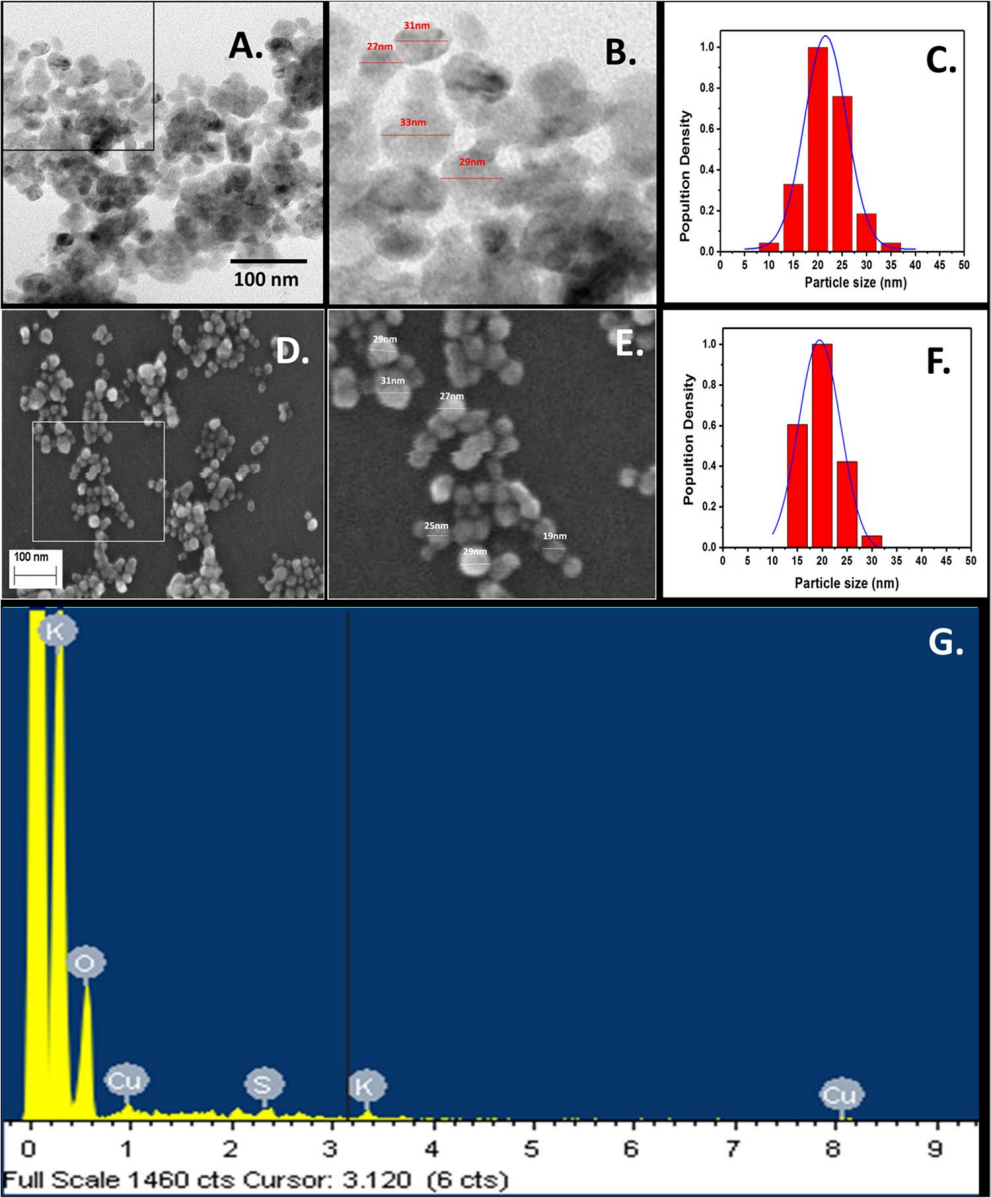
Size determination and validation of green synthesized CuO NP nanoparticles. (**A**) TEM (Scale bar of 100nm). (**B**) Magnified view of TEM. (**C**) Size distribution analysis. (**D**) FESEM (Scale bar of 100 nm). (**E**) Magnified view of FESEM. (**F**) Size distribution analysis. (**G**) EDS analysis of CuO NP.

**Figure 4:**
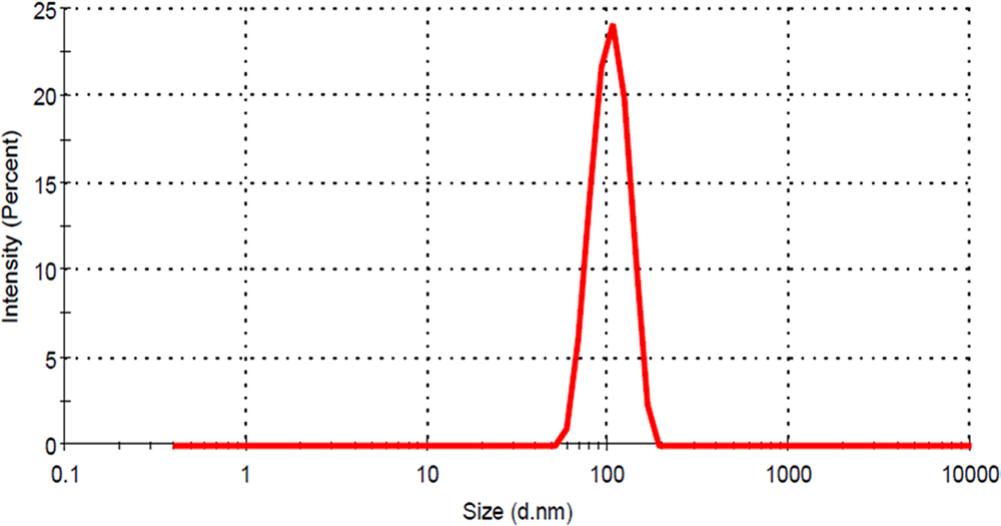
Size determination by hydrodynamic diameter measurement of green synthesized CuO NP nanoparticles by Dynamic light scattering.

Physiochemical characterization of synthesized CuO NP was done with standard characterization technique. As shown in Fig. 3A–C of TEM and Fig. 3D–F of FESEM, the particles were found to be spherical shape having a uniform size range of 25nm-35nm. EDS analysis confirmed the presence of CuO NP however small amount of carbon and silicon was also found in the experimental setup (Fig. 3G). As shown in Fig. 4, the Hydrodynamic diameter of freshly prepared suspension of synthesized CuO NP in HF buffer as determined by Zetasizer was found to be 102 ± 10 nm which indicated the presence of a uniformly distributed dispersion of CuO NP in the solution with attached water molecules around it. Determination of hydrodynamic diameter of synthesized CuO NP after 72 h in HF buffer showed the diameter of 109 ± 11 nm (Fig. S1) which confirmed that the particles were not getting aggregated in due course of time after 72 h.

### Determination of charge and optical properties of CuO NP

**Figure 5:**
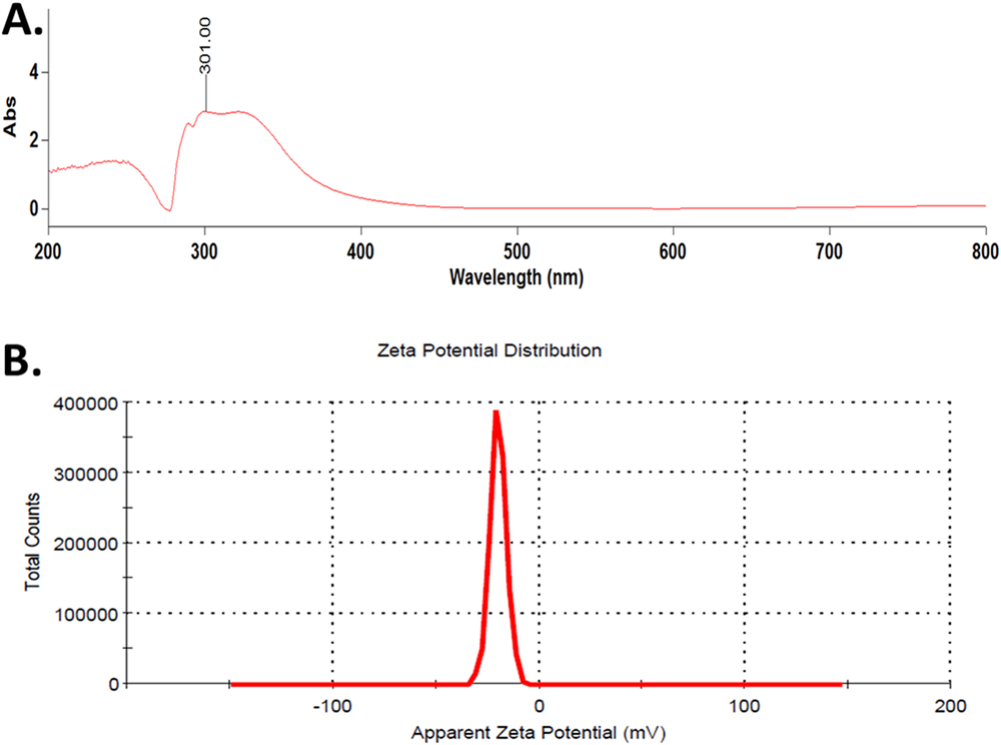
(**A**) UV-Vis spectrum of green synthesized CuO NP nanoparticles. (**B**) Zeta potential of CuO NP nanoparticles determined by Dynamic light scattering.

The optical property of synthesized CuO NP was determined by measuring the absorbance of light at a scanning range of 200 nm–800 nm in UV-Vis NIR spectrophotometer. As indicated by Fig. 5A, SPR peak at 301 nm was observed with two more peaks around 275–350 nm. SPR peak at 298 nm was observed in the CuO NP absorbance taken after 72 h as shown in Supplementary Fig. S2. Stability of CuO NP was evaluated by determination of zeta potential in the freshly prepared solution and after 72 h. As shown in Figs 5B and S3, the zeta potential of nanoparticles was found to be −34 ± 12 mV which remain significantly unchanged to −32 ± 14 mV after 72 h. The observation indicated the net negative charge at the surface of the synthesized nanoparticles and their stability in due course of time.

### XRD and FTIR analysis of green synthesized CuO NP

**Figure 6:**
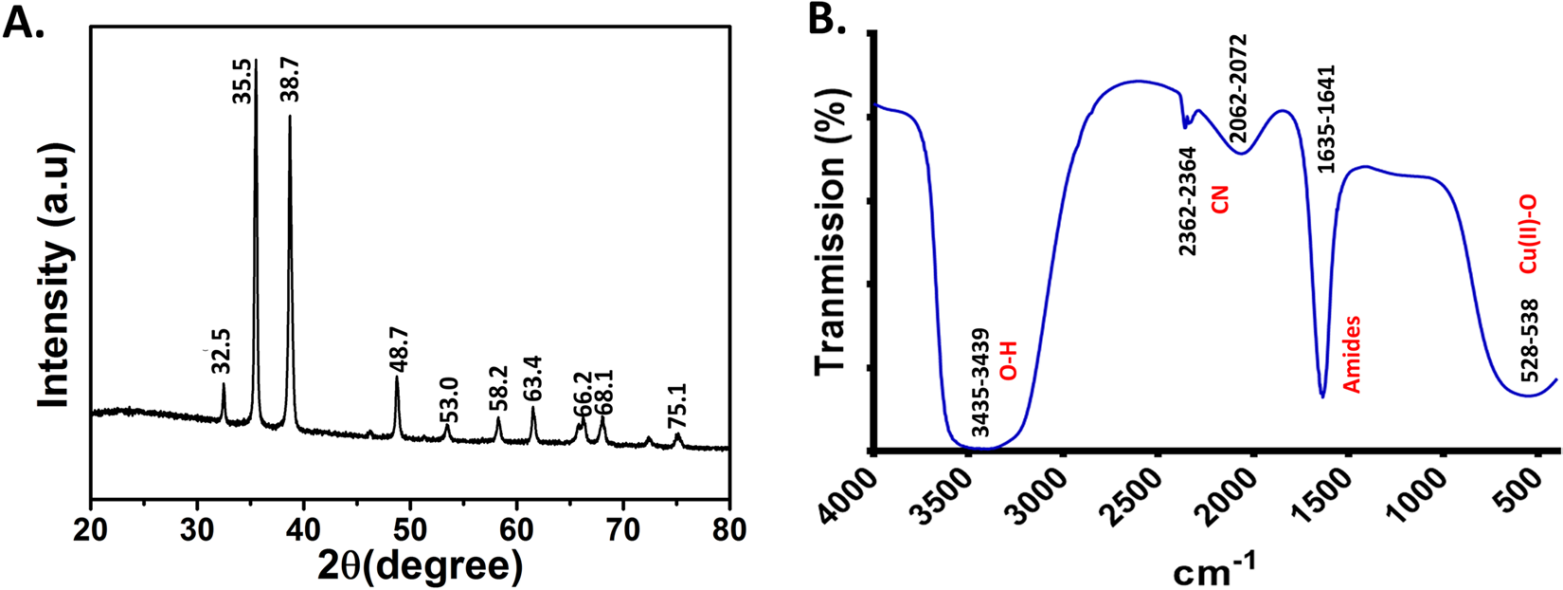
Physiochemical characterization of green synthesized CuO NP. (**A**) XRD analysis at 200 to 800. (**B**) FTIR spectrum at 500 cm^−1^ to 4000 cm^−1^.

Microcrystalline structure of green synthesized CuO NP was further analyzed using XRD technique. As shown in Fig. 6A, the characteristic XRD peak was observed at 32.5, 35.5, 38.7, 48.7, 53.0, 58.2, 63.4, 66.2, and 68.1 corresponding to 110, 002, 111, 202, 020, 202,113, 311 and 113 planes, respectively. The result indicated towards the formation of typical monoclinic CuO NP structure. Moreover, sharp peaks confirmed the highly crystalline nature of prepared CuO NP (JCPDS card no. 801268). Figure 6B showed the FTIR spectrum of synthesized CuO NP having band at 528–538 cm^−1^, 1635–1631 cm^−1^, 2062–2072 cm^−1^, 2362–2364 cm^−1^, and 3435–3439 cm^−1^. The broad peak observed at 3435–3439 cm^−1^ corresponds to the O–H and N–H bond stretching vibrations. The small peaks at 2362–2364 cm^−1^ and 2062–2072 cm^−1^ correspond to the stretching vibrations of the compounds containing C=N bonds. Sharp peak at 1635–1631 cm^−1^ denotes the presence of amide which may be due to the Calotropis floral extract biomolecules. Smaller peak found in the infrared spectrum at low frequencies below 700 cm^−1^ are due to Cu-O vibrations.

### *In vivo* toxicity of green synthesized CuO NP in Zebrafish embryo

**Figure 7:**
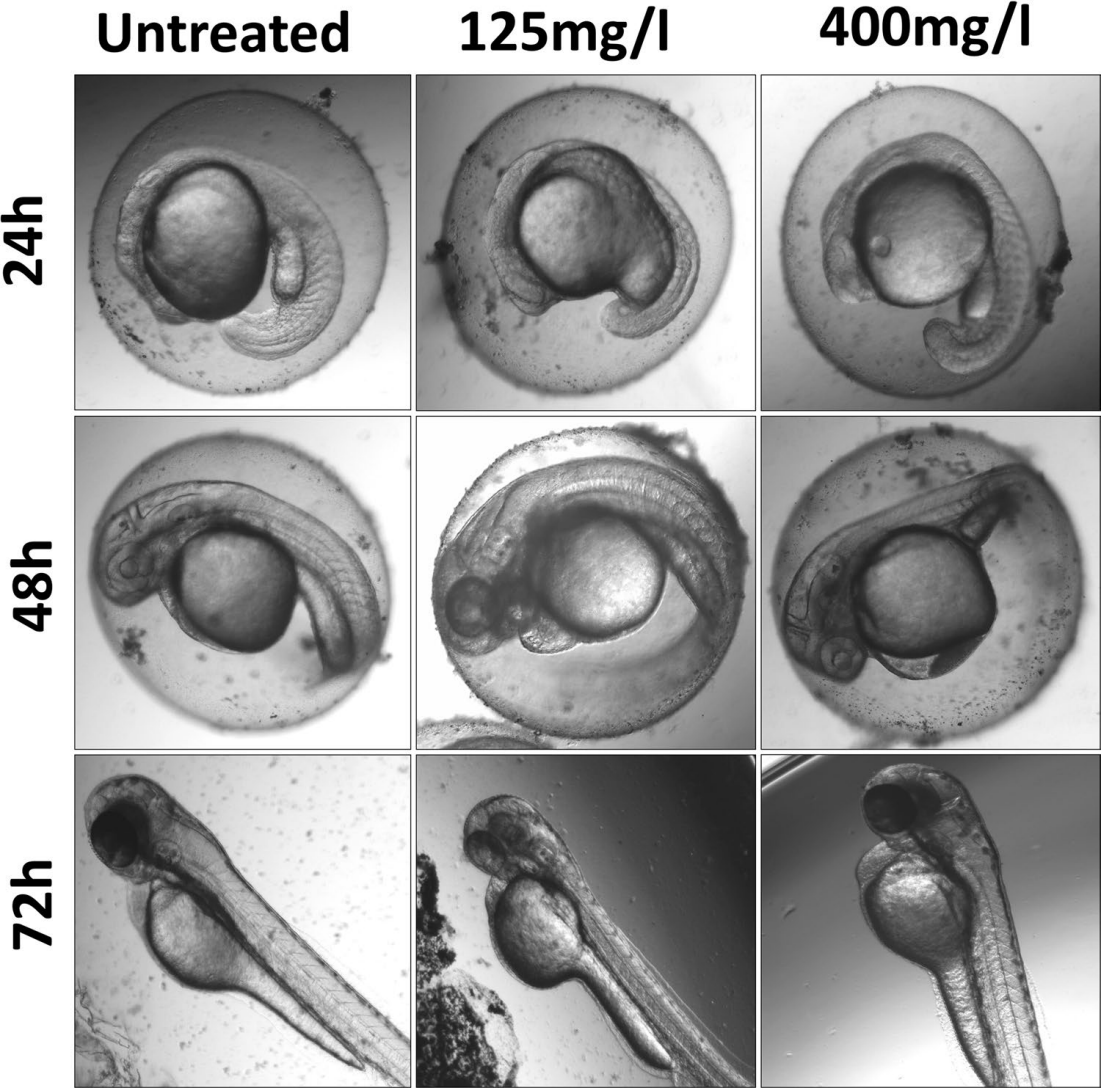
Bright field images of morphological analysis of Zebrafish embryos exposed to different concentration of green synthesized CuO NP.

**Figure 8:**
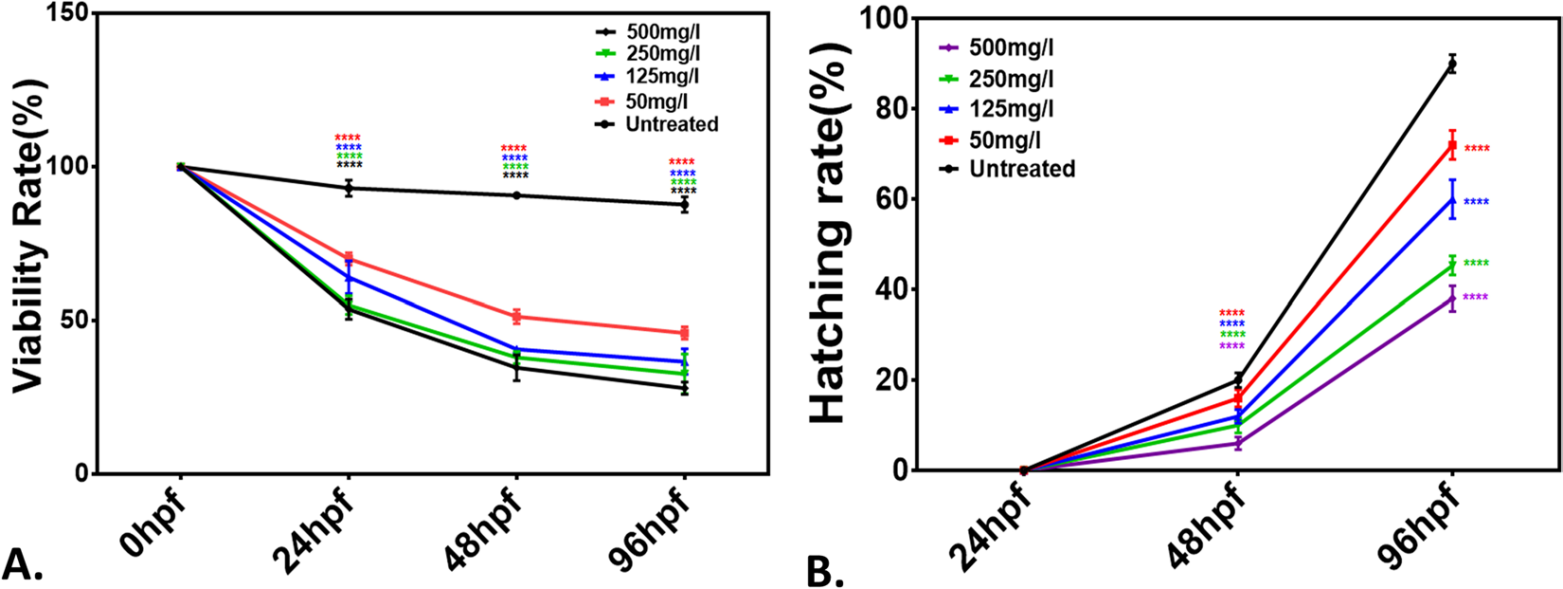
(**A**) Viability rate. (**B**) Hatching rate of Zebrafish embryos exposed to green synthesized CuO NP at different hours of post fertilization (hpf). All the measurements were taken in triplicate and the values were presented as mean ± SD of three independent experiments. *P < 0.05 denotes significant change from untreated embryos respectively as obtained from ANOVA analysis. Number of *presents the degree of significance.

The toxicity of green synthesized CuO NP was evaluated by determining their effect on physiological and morphological changes in Zebrafish embryo. The embryos were exposed to different concentration of synthesized and commercially purchased CuO NP and observed for their changes in comparison to the untreated embryos taken as a negative control of the experiment. Figure 7 shows the untreated and treated embryos at lower 125 mg/l and 400 mg/l concentration of green synthesized CuO NP. The concentration was chosen keeping in view of the LC50 calculated by measuring viability rate as shown in Fig. 8A. As revealed by Fig. 7, the nanoparticles were accumulated at the chorion and skin surface of 24, 48 and 72 hpf treated embryos respectively. In 24 hpf embryos, the yolk sac was found to be swelled while at higher concentration there was an indication of notochord bending. At 48 h exposure, pericardial edema was observed both at a low and high concentration with higher acute results in higher concentration. Abnormal abdominal development was identified in 72 hpf at 125 mg/l exposed embryos which were accompanied by pericardial edema and poorly developed and stiffened notochord at 400 mg/l.

To calculate and compare the minimum concentration of green synthesized and commercially purchased CuO NP having 50% viability (LC50) of exposed embryos, viability rate was determined in a group of 20. It was calculated as the percentage of viable embryos and larvae in total number of treated embryos. As shown in Figs 8A and S4A the viability rate was found to be reduced with an increase in concentration and exposure time of both types of CuO NP. LC50 was found at 175 ± 10 mg/l concentration for green synthesized CuO NP while it was 45 ± 10 mg/l in case of commercially purchased CuO NP. Hatching rate was also calculated as the percentage of hatched embryos in the group of total exposed embryos. Figures 8B and S4B shows the hatching rate of untreated and treated embryos with synthesized and commercial CuO NP. It was found to be dependent on concentration and exposure time. Interestingly, hatching rate was found to be higher in case of embryos exposed to green synthesized CuO NP as compared to commercial one.

### Oxidative stress and Apoptosis analysis in Zebrafish embryos exposed to CuO NP

**Figure 9:**
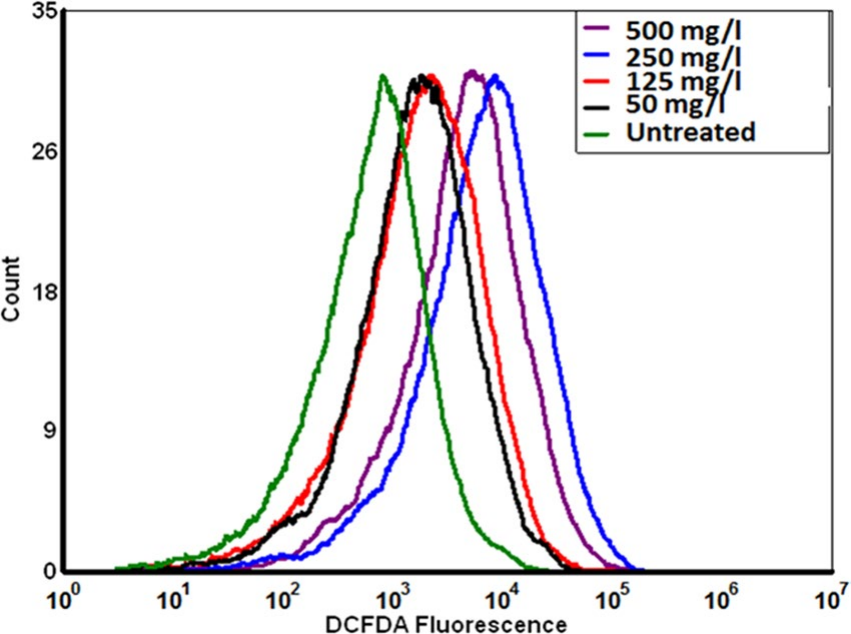
ROS level measured by DCFDA fluorescence level of Zebrafish embryos exposed to different concentration of green synthesized CuO NP.

To determine and compare the effect of synthesized and commercial CuO NP in Zebrafish embryos at the cellular level, induction of ROS and apoptosis was analyzed in embryos after 72 hpf of treatment with the help of flow cytometry and fluorescent microscopy. ROS determination was done with the help of DCFDA staining in Zebrafish embryo cell suspension after treatment. CuO NP suspension in HF medium was incubated with DCFDA stain to check any interference of the nanoparticles. As shown in Figs 9 and 5, the green fluorescent intensity of DCFDA was found to be right shifted depicting the increase in ROS induction with increase in the concentration of CuO NP in both cases. However, the increment was higher in embryos cells treated with commercial CuO NP as compared to green synthesized CuO NP. No significant interference in DCF fluorescence was observed in presence of only CuO NP as shown in Fig. 6. Further, analysis of consequences of ROS production was done by checking the level of apoptosis in cells of Zebrafish larvae exposed to lower (125 mg/l) and higher (400 mg/l) concentration of CuO NP. The concentrations were chosen in view of LC50 determined by viability assay.

**Figure 10:**
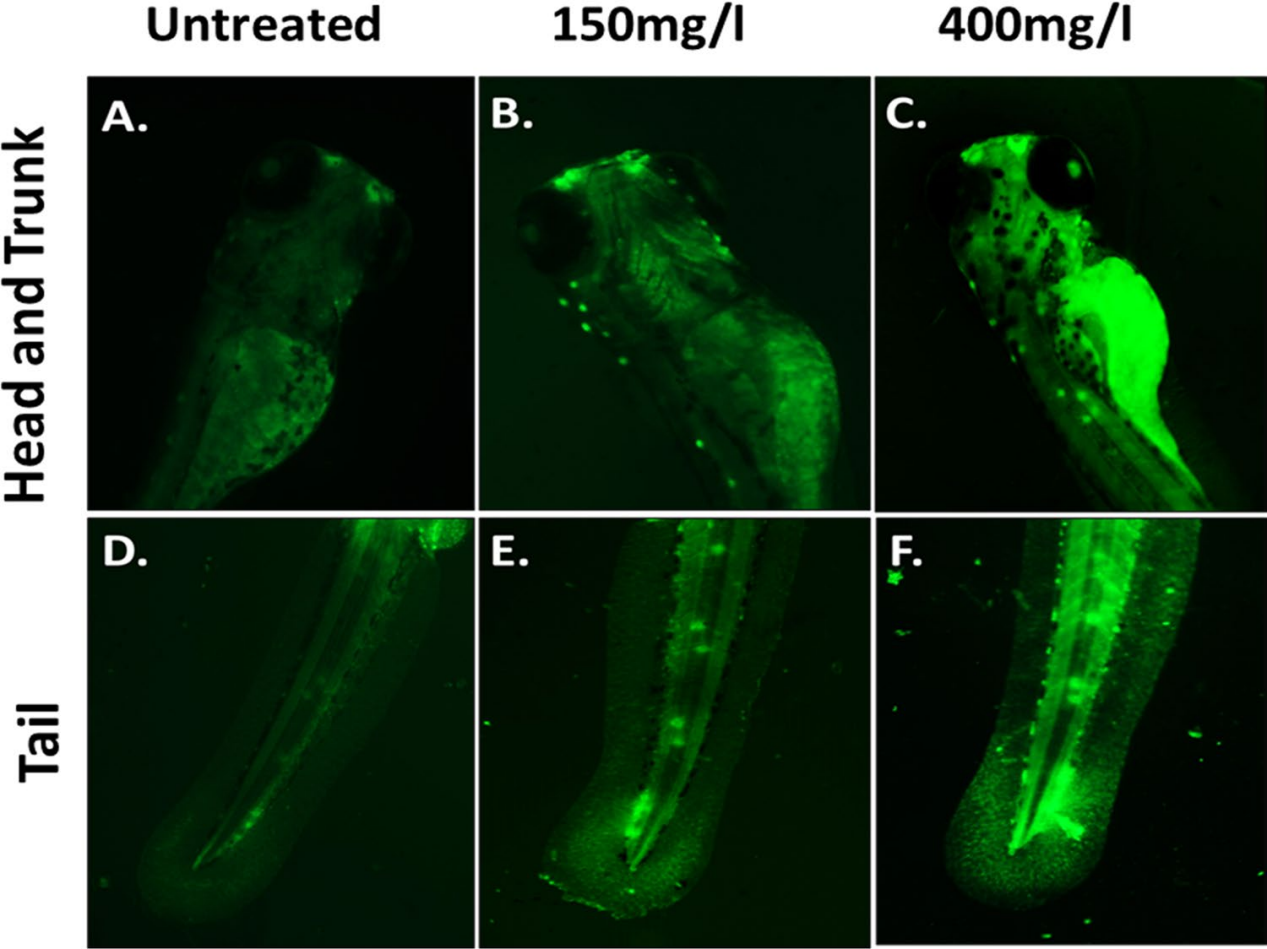
Apoptosis of Zebrafish larva cells (96 hpf) exposed to different concentration of green synthesized CuO NP as determined by Acridine orange (AO) staining.

**Figure 11:**
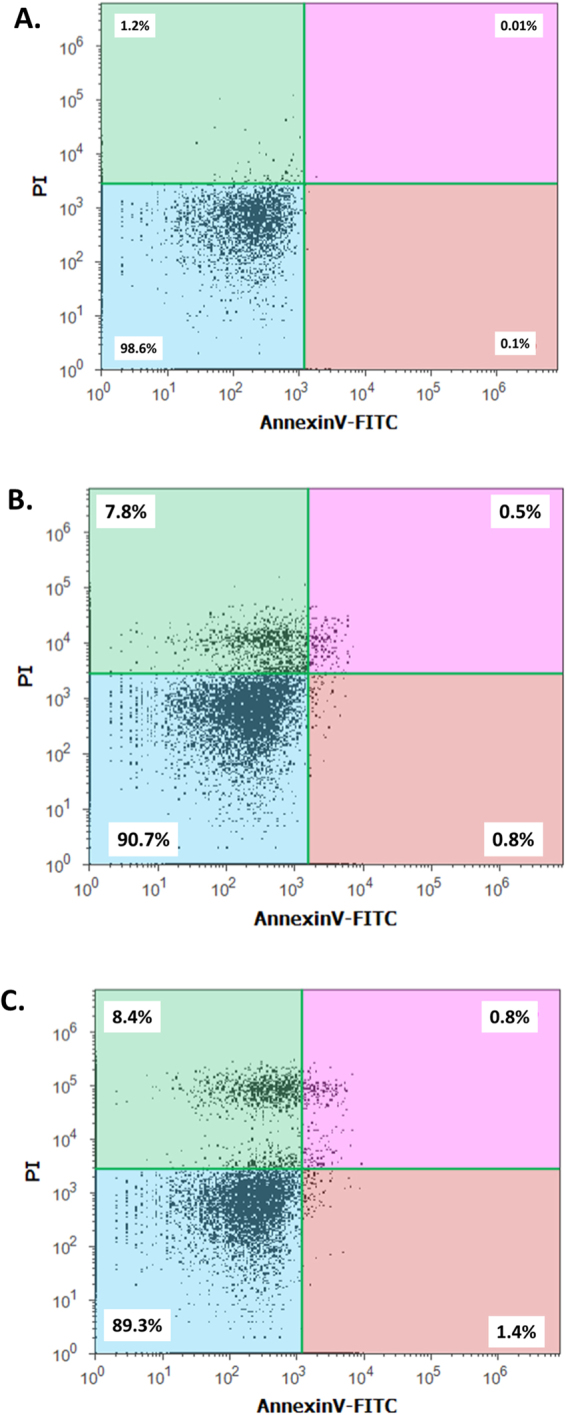
A poptosis of Zebrafish larva cells (96 hpf) exposed to different concentration of green synthesized CuO NP as determined by flow cytometry analysis by AnnexinV-FITC/PI assay. (**A**) Untreated. (**B**) 150 mg/l exposed embryos. (**C**) 400 mg/l exposed embryos.

Cellular apoptosis was identified with the help of Acridine orange staining as shown in Figs 10 and 7. The increase in green patches was clearly identified in both head and trunk region of Zebrafish larvae exposed to 150 mg/l of synthesized and commercial CuO NP. Moreover, the fluorescent intensity and the patches were found to be highly intensified at 400 mg/l of exposure in both head and tail region. Interestingly, the fluorescent intensity was more intense in embryos exposed to commercial CuO NP as compared to green synthesized CuO NP. Further quantitative estimation of cellular apoptosis was done using Annexin V/FITC-PI assay by flow cytometry. As shown in Fig. 11, percentage of apoptotic cells was found to be 1.3 and 2.2% in embryonic cells exposed to 150 mg/l and 400 mg/l of synthesized CuO NP while the numbers of apoptotic cells were found less (0.13 and 0.3%) in embryos exposed to same concentration of commercial CuO NP (Fig. S8). Interestingly, the percentages of necrotic cells were higher (7.8% and 26.9%) compared to 7.8% and 8.4% in embryos exposed to 150 mg/l and 400 mg/l of commercial CuO and synthesized CuO NP depicting higher and severe cell toxicity in case of commercial CuO NP. The result revealed the dependence of cellular apoptosis with ROS induction in Zebrafish embryos on exposure of CuO NP and supported the fact of green synthesized CuO NP being biocompatible as compared to commercial CuO NP.

### *In silico* analysis of CuO nanoparticles interaction

**Figure 12:**
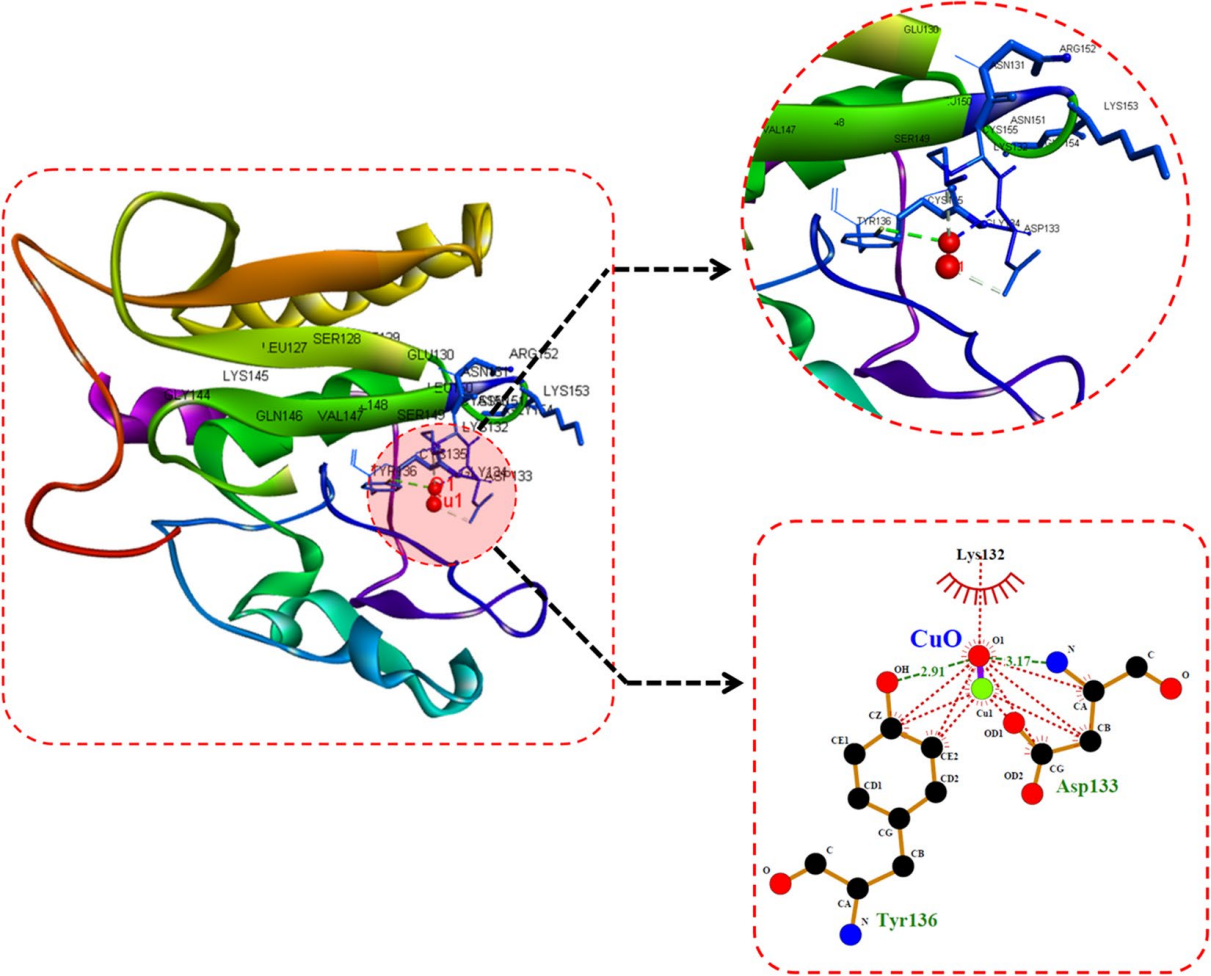
Molecular docking analyses of he1a with CuO nanoparticle showing interacting residues using LigPlot+ and Discovery studio Visualizer.

**Figure 13:**
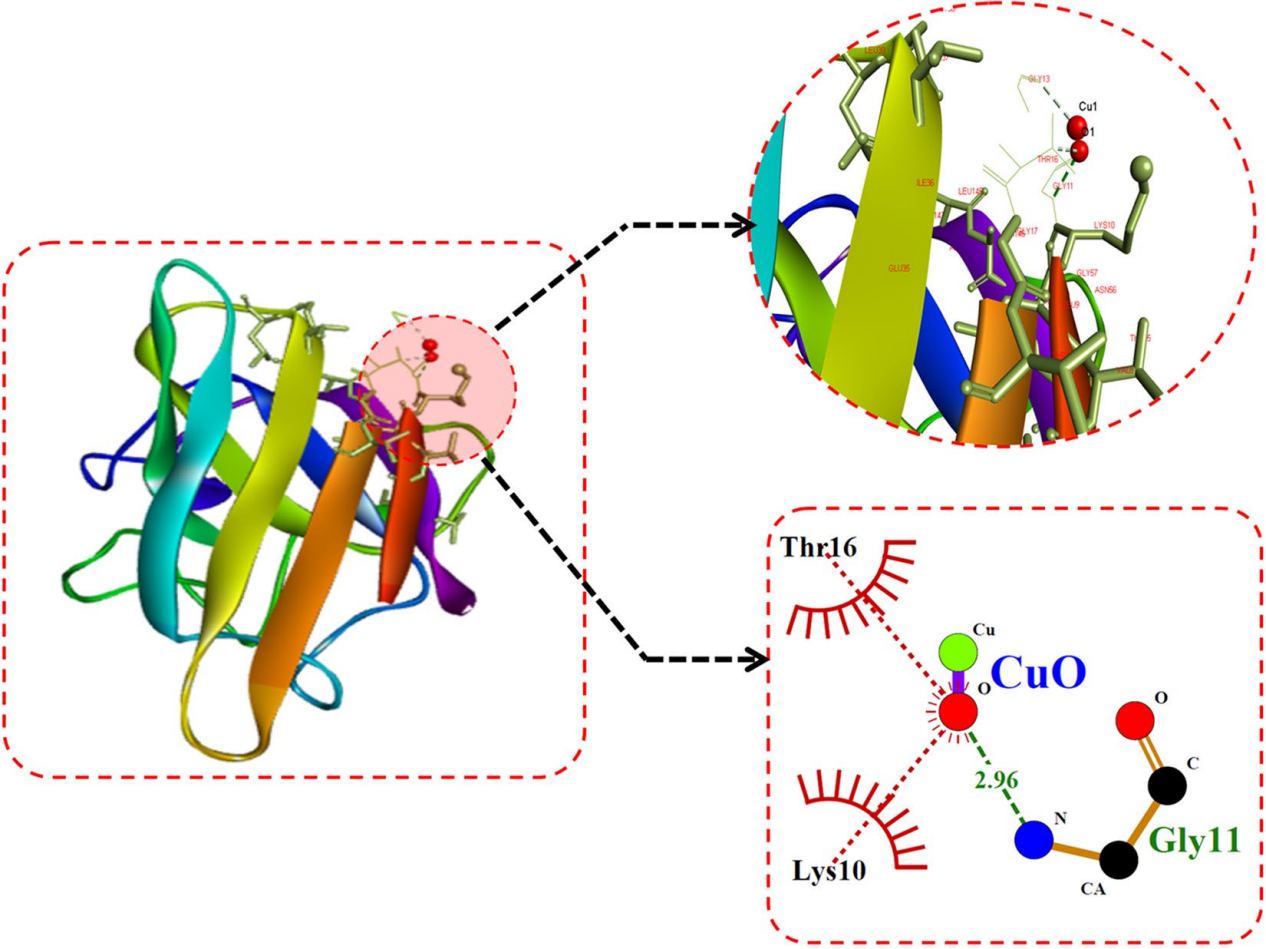
Molecular docking analyses of sod1 with CuO nanoparticle showing interacting residues using LigPlot+ and Discovery studio Visualizer.

**Figure 14:**
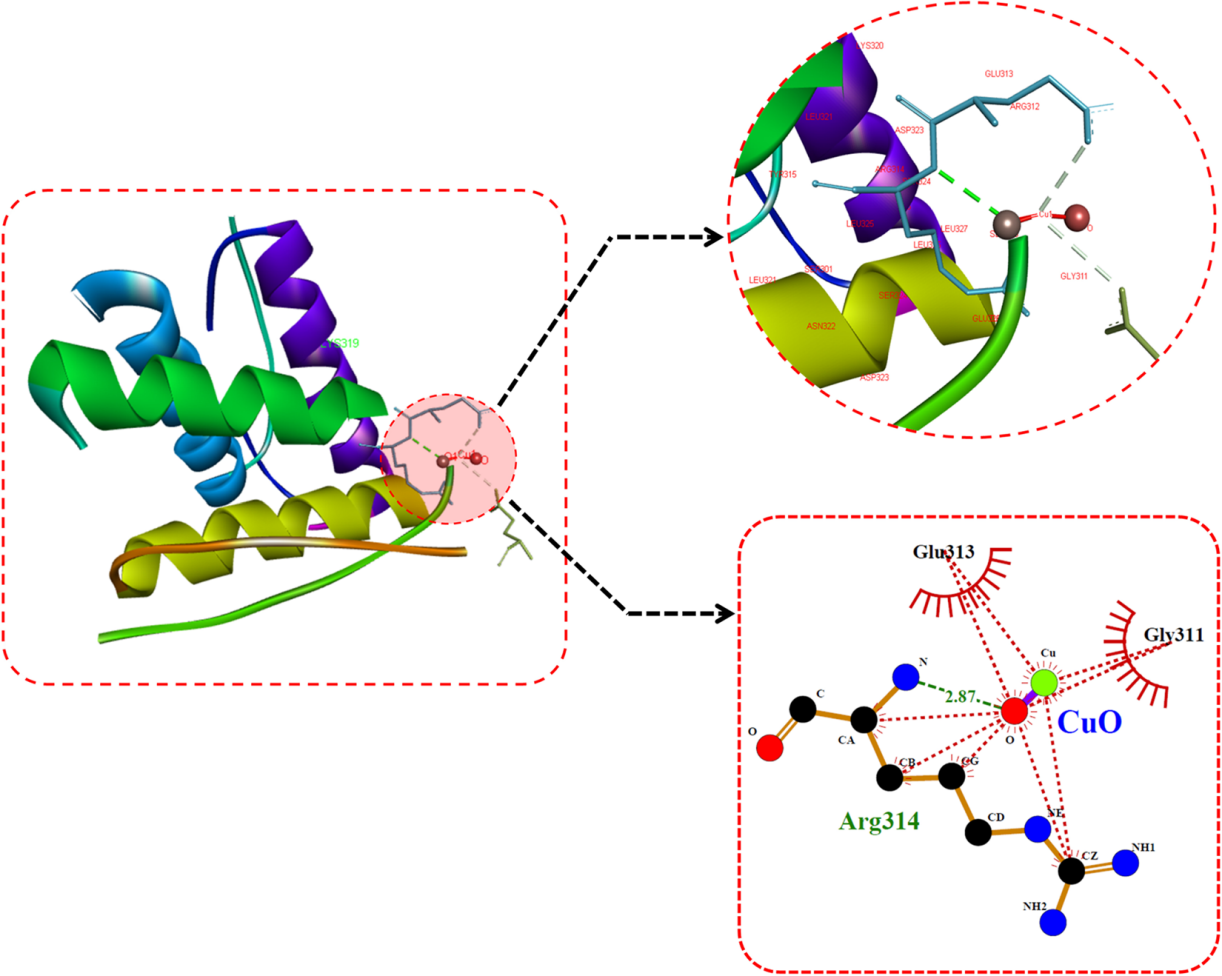
Molecular docking analyses of p53 with CuO nanoparticle showing interacting residues using LigPlot+ and Discovery studio Visualizer.

**Figure 15:**
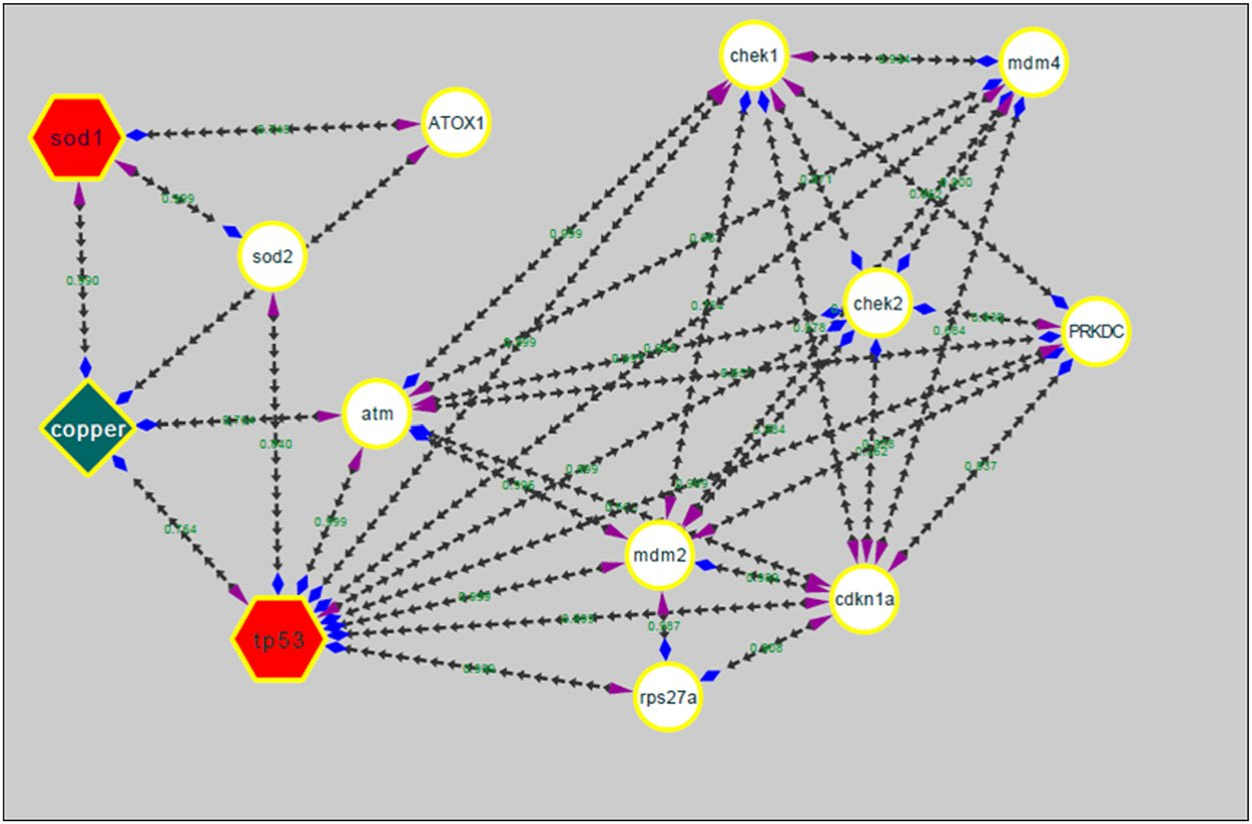
Pathway showing CuO interaction mechanism involving sod1 and tp53 proteins derived from STITCH and analyzed using Cytoscape.

For investigation of CuO nanoparticles interaction with Zebrafish embryo and their effect on hatching at the molecular level, *in silico* approach was engaged. Molecular docking analysis of hatching enzyme (he1a) with CuO nanoparticles showed hydrophobic as well as hydrogen bond interaction with different amino acid residues. As shown in Fig. 12 and Table 2, the hydrophobic interaction of CuO nanoparticles with Aspartic acid (Asp), lysine (Lys), and tyrosine (Tyr) residues was predicted by Autodock. While Hydrogen bond interaction was observed with Aspartic acid (Asp) and tyrosine (Tyr) residues. 2D Ligplot presentation showed H-bond length of 2.91 Å and 3.17 Å with Tyrosine and Aspartic acid residues respectively. To investigate the molecular mechanism of induction of oxidative stress in Zebrafish embryos by CuO NP, analysis of the interaction of the Sod1 enzyme with them was studied through *in silico* approach.As shown in Fig. 13, CuO NP was found to have an hydrophobic interaction with Sod1 via Threonine (Thr) and lysine (Lys) residues. A firm hydrogen bond interaction with a bond length of 2.96 Å was found with Gysine (Gly) residue. Further, concomitant elucidation of experimental observation of apoptosis was performed by molecular docking with the p53 enzyme with CuO NP. Figure 14 showed the molecular interaction of CuO NP with p53 displaying hydrophobic interaction with glutamic acid (Glu) and Lysine (Lys) along with a hydrogen bond interaction with Arginine (Arg) with a bond length 2.87 Å. Intermolecular communication of different enzymes in CuO NP influence was depicted by pathway investigation through STITCH^21^. As displayed in Fig. 15, CuO NP was influencing sod1and p53 by means of ATOX and sod2 enzymes. Some other enzymes were also found to be linked to pathways like atm, mdm, and chek.

**Table 2:** Molecular docking energies of CuO nanoparticle interaction with different receptor protein using Autodock showing binding modes and energies in kcal/mol.

Receptors	Conformation	Binding Energies (Kcal/mol)	Binding Residues	H-Bond Distance	Atoms Involved
He1a	3	−1.23	Tyr 136	2.91	O1-OH
			Asp 133	3.17	O1-N
Sod1	3	−0.91	Gly 11	2.96	O1-N
Tp53	5	−1.07	Arg 314	2.87	O1-N

Total binding energy = vdW + Hbond + desolv Energy − Electrostatic Energy.

## Discussion

With the increase in demand and production of CuO nanoparticles, their exposures to the ecosystem and human society have also intensified which may lead to the adverse and lethal effects. CuO NP available commercially is prepared from chemical or physical synthesis route which are having the issue of high cytotoxicity and environmental hazards. Green synthesis has been recognized as a potential solution of this problem^22^. In this study, Synthesis of CuO nanoparticles have been done by green route of synthesis using medicinal plant extract and their *in vivo* impact on Zebrafish embryos were assessed. Floral part of *Calotropis gigantea* has been well recognized for its medicinal properties worldwide. Moreover, their availability is known to be cosmopolitan^23^. Aqueous extract of floral part of *Calotropis gigantea* was used to synthesize CuO NP (Fig. 1). Formation of black color and precipitate at the end of the incubation confirmed the synthesis of CuO nanoparticles. Faint bluish green color with white precipitate during initial reaction indicated towards the formation of intermediates like Cu(II) hydroxide (Cu(OH)_2_). The extract was found to contain different biomolecules as revealed by GCMS analysis (Fig. 2, Table 1). Comparison of chromatogram of extract before and after synthesis of CuO NP depicted the utilization of compounds containing –OH for synthesis. Previous literatures have also reported the presence of different biomolecules like flavonol glycosides^24^, cardenolides^25^, saccharides, lipids, and DNA from different parts of *Calotropis gigantea* ^26,27^. Literatures have also reported presence of different types of proteins like Calotropin^28^ and different types of protease in *Calotropis* ^29^. With reference to the experimental results and literature reports, these biomolecules can be attributed for the reduction and stabilization of CuO NP for their synthesis from Copper chloride salt. The green synthesized nanoparticles were characterized by different techniques. Nanosize of the CuO NP was found to be in the range of 25–35 nm as determined by FESEM and TEM image (Fig. 3A,B). The validation of CuO NP presence was done by EDS analysis. Presence of Cu and oxygen validated the formation of CuO NP in the reaction setup however small amount of Silicon was also found which may be due to the silicon chip used for the substrate in the analysis. The synthesis of CuO NP from copper chloride salt can be depicted as:

$$\begin{array}{c}{\bf{CuC}}{{\bf{l}}}_{{\bf{2}}}.{\bf{2}}{{\bf{H}}}_{{\bf{2}}}{\bf{O}}+{\bf{2}}({\bf{Biomolecules}}){\bf{OH}}\to {\bf{Cu}}{({\bf{OH}})}_{{\bf{2}}}+{\bf{2}}({\bf{Biomolecule}}){\bf{Cl}}+{\bf{4}}{{\bf{H}}}_{{\bf{2}}}{\bf{O}}\\ \quad \quad \quad \quad \quad \quad \quad {\bf{Cu}}{({\bf{OH}})}_{{\bf{2}}}\to {\bf{CuO}}{\bf{NP}}\,({\bf{Capped}}\,{\bf{and}}\,{\bf{stabilized}})+{{\bf{H}}}_{{\bf{2}}}{\bf{O}}\end{array}$$

Size of the nanoparticles varies with the solvent medium because of attachment of solvent molecules with them. Cytotoxicity effect also varies with the variation of the size in the medium^30,31^. Estimation of hydrodynamic diameter reveals the size of a particle in the medium. As indicated in Fig. 3, the hydrodynamic diameter of synthesized CuO NP was found to 102 ± 10 nm which was not changing with due course of time in 72 h. Results confirmed the polydispersity of nanoparticles in HF medium. The variation of the size as compared to the size determined by FESEM can be attributed to the attachment of water molecules with the CuO NP in an aqueous medium. Optical properties are regarded as one of the important parameters to determine the nature of a nanoparticle. The absorbance of light by a particle determines its nature and stability which can be calculated by the surface plasmon resonance peak (SPR).Gans modification of Mie theory predicted the alteration from spherical geometry of a particle on shifting of SPR peak. Synthesized CuO NP possessed SPR peak at 301 nm showing a slight deviation from spherical geometry of the nanoparticles (Fig. 5A). SPR peak was not found to deviate with time indicating the optical stability of synthesized CuO nanoparticles with time. It is important to investigate the stability of nanoparticles in a medium to assess its complementary effect^32^. Agglomeration and stability depend on the charge of the nanoparticles^33^ which can be determined by estimation of their Zeta potential. Zeta potential can be defined as a function of electrophoretic mobility of molecules in the medium^32^ and can be regarded as a parameter of the stability of particles in the medium. Zeta potential of as synthesized CuO NP was found to be −34 ± 12 mV (Fig. 5B) in fresh suspension and was −32 ± 14 nm after 72 h suspension. Our results were in line with previous reports of synthesized CuO NP by other methods and were confirming the stability of synthesized CuO NP^14^.

Nanotoxicity of nanoparticles with *in vivo* system can be defined as the effective change in developmental, physiological and morphological level in the animal model due to exposure to the nanoparticles. The changes in the developmental and morphological level were assessed for commercial and synthesized CuO NP in Zebrafish embryos at different concentration. Morphological changes were observed at 24, 48 and 72 hpf of Zebrafish embryos on exposure of CuO NP at a low level. At a low concentration exposure in 24 and 48 hpf, the yolk sac was abnormal with pericardial edema while at higher concentration the developing notochord was found to bent (Fig. 7). Similarly, at 72 hpf, less development of abdominal cavity was detected at a lower concentration of CuO NP exposure while a clear indication of less developed notochord was observed at higher concentration. The observation can be correlated with the calculation of viability rate and hatching rate of embryos exposed to different concentration of synthesized CuO NP. The viability and hatching rate of embryos were concentration dependent and were significantly varied in commercial and green synthesized CuO NP exposure. Interestingly, both were found higher in case of synthesized CuO NP indicating towards having more biocompatibility as compared to commercial CuO NP. The reduced hatching rate of embryos (Fig. 8B) can be attributed to the less development of chorion at higher concentration^34,35^. Interaction of CuO NP with the hatching enzyme molecules can also be one of the basic reasons. ZnO, silver and gold nanoparticles have been reported to lower down the hatching rate because of their interaction with hatching enzyme and lowering down their activity^34^. Interference of CuO NP in the hatching of embryos can be reasoned for the increase in mortality rate of embryos with the concentration. Synthesized CuO NP nanoparticles showed LC50 of 175 ± 10 mg/l which was quite high as compared to the LC50 of commercial CuO NP. Hence, the biocompatibility of synthesized CuO NP can be argued against the conventional prepared CuO NP.

Cytotoxicity of the nanoparticles subsists due to cellular physiological changes like oxidative stress and apoptosis. Induction of oxidative stress has been regarded as one of the important regulatory factors of cytotoxicity of a nanoparticle. CuO nanoparticles have been reported to induce oxidative stress by the production of ROS in different cell lines^4^. *In vivo* cytotoxicity studies of CuO nanoparticles in the mouse model have also reported changes in oxidative stress level in different organs like heart, liver, and spleen^1,36^. Commercially available CuO NP have also shown production of oxidative stress by upregulation of ROS level leading to teratogenicity and genotoxicity in Zebrafish embryos^14^. To substantiate and compare the effect of green synthesized CuO NP, the oxidative stress level was measured in Zebrafish embryos exposed to CuO NP at different concentration of commercial and green synthesized CuO NP. Induction and production of ROS were found to be varying with concentration (Fig. 9) and were higher in case of commercial CuO Np exposure. Enhancement of ROS level due to exposure of CuO NP can be attributed to the internalization of CuO NP inside Zebrafish embryos and larva. It may be the possibility of chorion and skin pores clogging due to entrapment of CuO NP leading to hypoxic condition inside the embryo and larvae. This hypoxic condition can be correlated to the increased level of ROS in order to neutralize the physiology of mitochondrial respiratory system. Generation of ROS lead to a lethal effect on cells and can increase the apoptosis process. To validate this hypothesis, apoptosis of Zebrafish cells were analyzed experimentally. The numbers of apoptotic and necrotic cells were found to be increased with concentration as depicted by the enhanced fluorescence intensity of Acridine orange stain and increased percentage of apoptotic and necrotic cell indicated by AnnexinV-FITC/PI staining in Zebrafish tissues (Figs 10,11). Moreover, the intensity of necrosis was higher in commercial CuO NP exposure as compared to green synthesized CuO NP at the same concentration. The experimental results can be well supported by the fact that with the exposure of CuO NP, clogging or interference in protein level occurs which causes the enhanced cell apoptosis and necrosis as a consequence of induction of oxidative stress in Zebrafish embryonic cells and it was higher in case of commercial CuO NP. The computational analysis further elucidated the molecular mechanism of CuO NP depicting variable bond interaction with he1a, Sod 1and p53 enzymes. It can be interpreted from the molecular docking analysis that retardation of hatching due to CuO NP exposure was a result of the influential functionality of he1a enzyme leading towards their inhibition. He1a enzyme has been reported as a key factor in hatching phenomenon^37^. These molecular changes lead to the phenotypical expression of concentration dependent change in hatching and mortality rate of Zebrafish embryos. Expression of Sod1 and p53 has been reported having a key role in the induction of oxidative stress due to ROS production^38^ and apoptosis^39^. With reference to computational analysis in concomitance with experimental results, a molecular aspect of CuO NP cytotoxicity can be attributed to the abnormal functionality of sod1 and p53 enzyme due to hydrogen bond interaction with different amino acid residues influencing each other. The influential functionality of enzymes due to CuO NP can be assumed for other proteins playing part in physiological process as depicted by pathway analysis (Fig. 15).

These studies at the cellular and embryonic level investigated the estimated cytotoxic effect of CuO NP in development and physiology of Zebrafish and depicted the importance of green synthesized CuO NP in comparison to commercial CuO NP.

## Conclusion

In summary, successful green synthesis of stable, well characterized CuO nanoparticle was done and their *in vivo* cytotoxic impact was investigated with comparison to commercial available CuO NP. Data from this report suggested a new novel method of green synthesis of CuO nanoparticles. Cytotoxicity studies on Zebrafish embryos revealed that the observed nanotoxicity of CuO nanoparticles is mediated by ROS level. Short term exposure of green synthesized CuO NP got accumulated at the surface of embryos triggering the generation of ROS inducing imbalance leading to oxidative stress. The antioxidant mechanism fails to cope with the increased oxidative stress due to the abnormal functionality of responsible proteins (sod1, p53) leading to apoptosis and necrosis of cells. Apoptosis and necrosis in cells of embryos which are unusual for developing cells led to malformation of organs and ultimately death. Hence, the release of CuO NP in the environment can create a serious effect on growth and generations of the aquatic ecosystem. Synthesis and utilization of green synthesized CuO NP can be a potential solution for this with condition provided that they are used in optimal concentration. These findings will intend the metal oxide nanoparticle producers to look for green synthesis as an alternative. Moreover, these findings will pave the path for further research in CuO nanotoxicity to Zebrafish at the molecular level.

## Electronic supplementary material

Supplementry Information
