# Supplementary material for: Mechanistic insight to ROS and Apoptosis regulated cytotoxicity inferred by Green synthesized CuO nanoparticles from Calotropis gigantea to Embryonic Zebrafish
Source: Sci Rep. 2017 Nov 24;7:16284. doi: 10.1038/s41598-017-16581-1 (PMC5701131; doi:10.1038/s41598-017-16581-1)
Supplement: Supplementary file 1 — Supplementry Information [file 41598_2017_16581_MOESM1_ESM.pdf]

Mechanistic insight to ROS and Apoptosis regulated cytotoxicity inferred by Green synthesized CuO nanoparticles from *Calotropis gigentea* to embryonic zebrafish.

Puja Kumari[1ꜜ], Pritam Kumar Panda[2ꜜ], Ealisha Jha [3], Khushboo Kumari[1], Kumari Nisha[1],

M. Anwar Mallick[1]*, Suresh K Verma[4]*

sureshverma22@gmail.com , amallick1@rediffmail.com

Supplementary Information

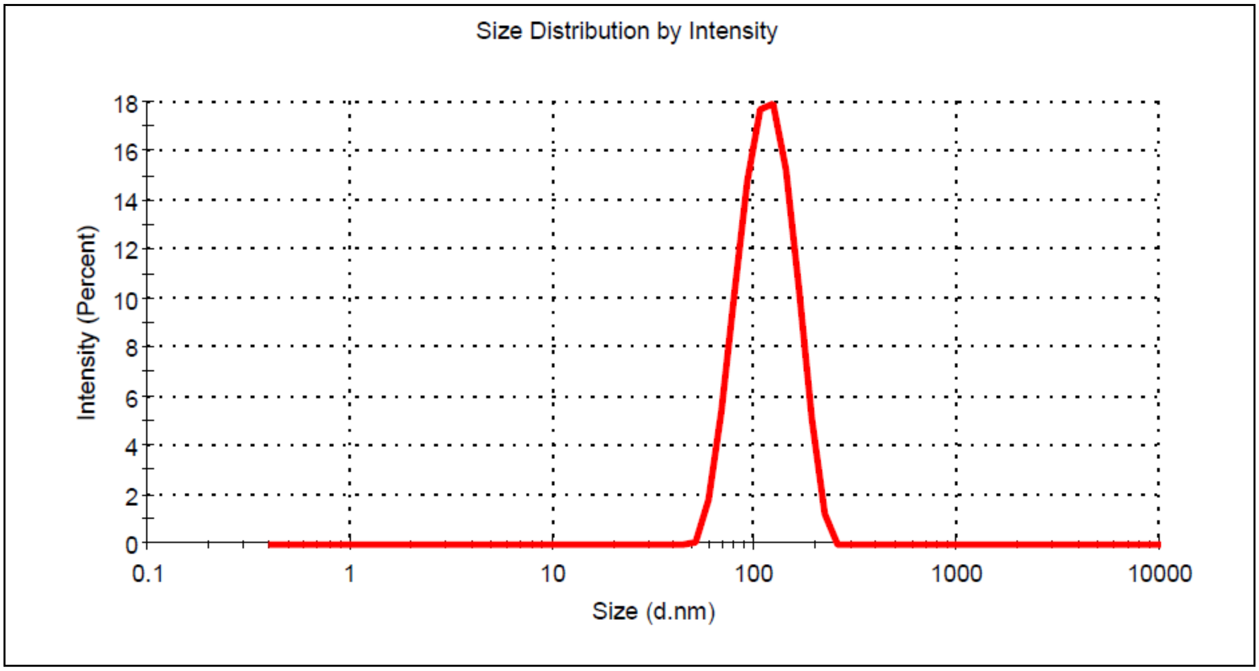

Figure S1: Hydrodynamic diameter of green synthesized CuO NP in 72h prepared suspension in HF medium.

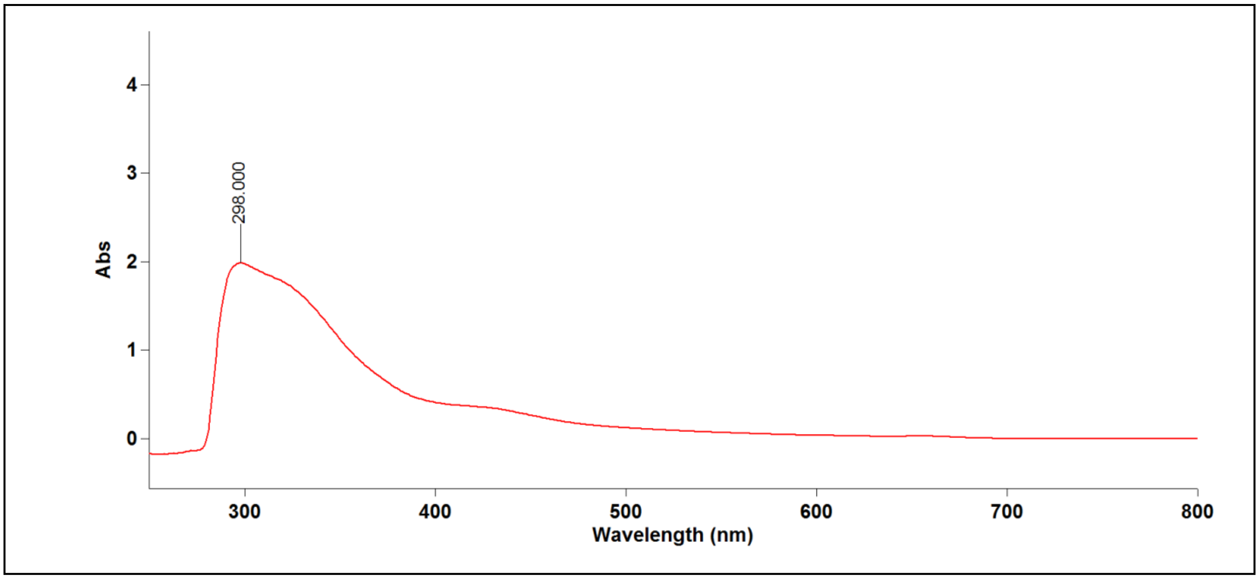

Figure S2: SPR peak of 72h suspension of green synthesized CuO NP as determined by UV-Visible spectrophotometry spectrum taken at a range of 200nm-800nm.

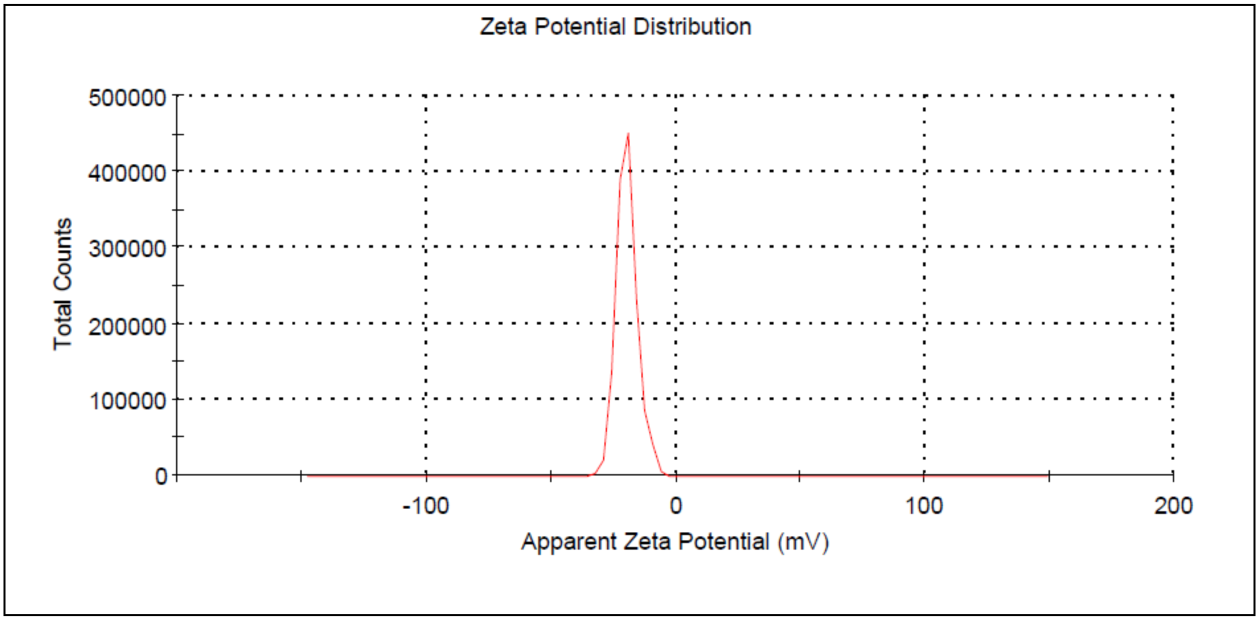

Figure S3: Zeta potential of green synthesized CuO NP in 72h prepared suspension in HF medium.

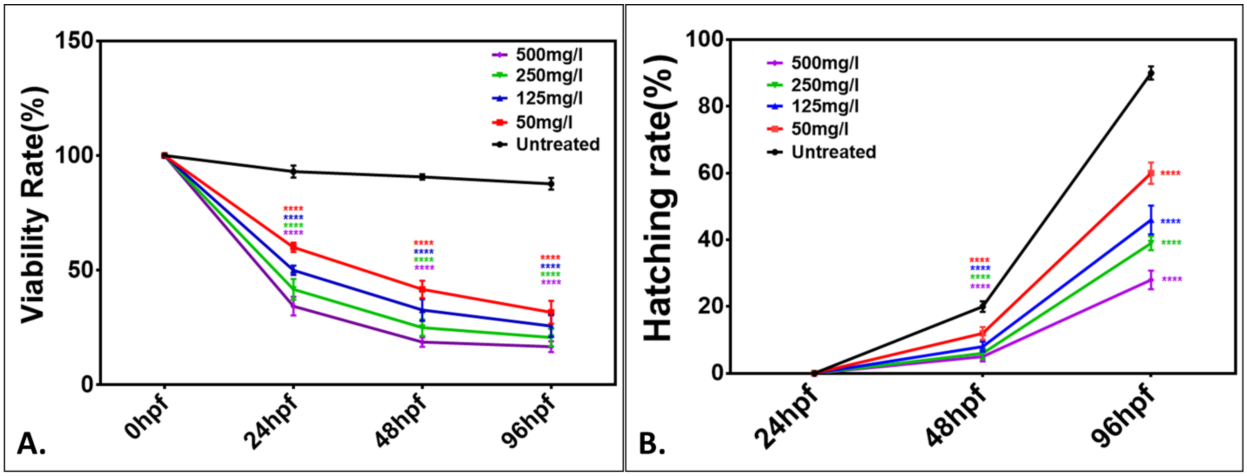

Figure S4: (A) Viability rate (B) Hatching rate of Zebrafish embryos exposed to commercial CuO NP at different hours of post fertilization (hpf).

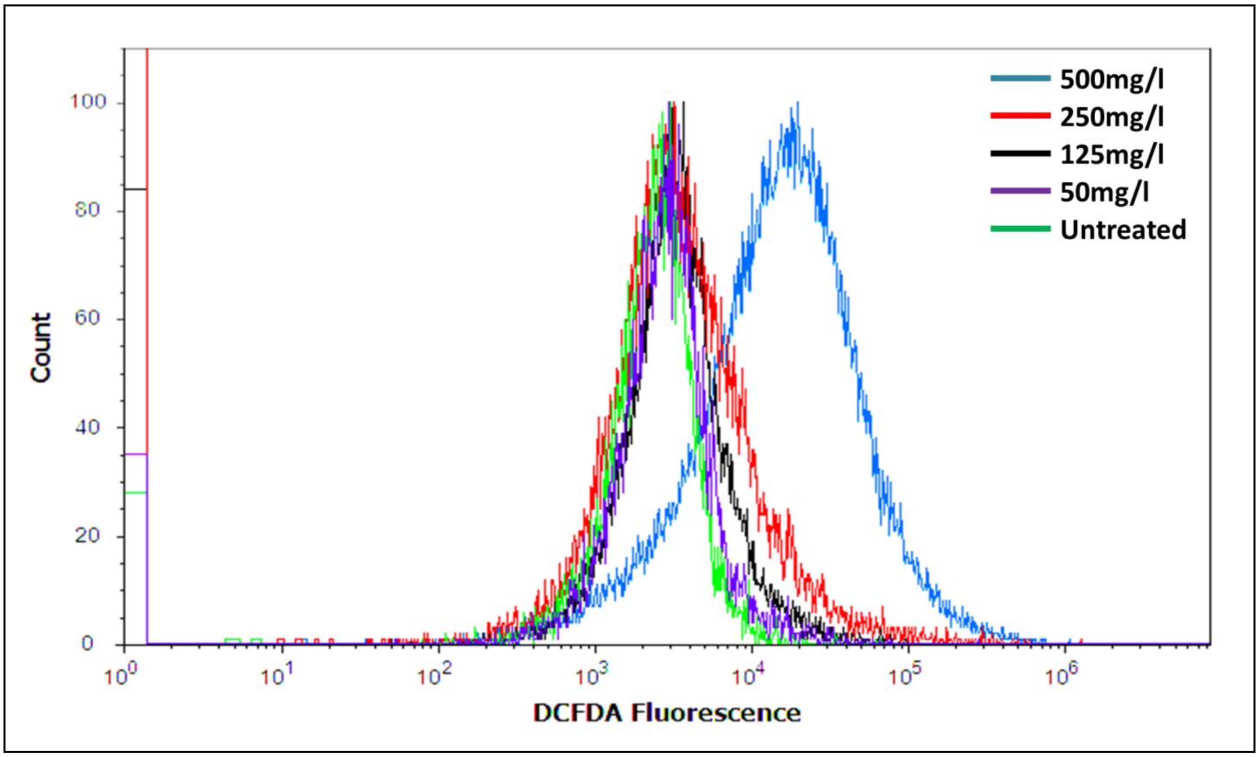

Figure S5: ROS level measured by DCFDA fluorescence level of zebrafish embryos exposed to different concentration of commercial CuO NP.

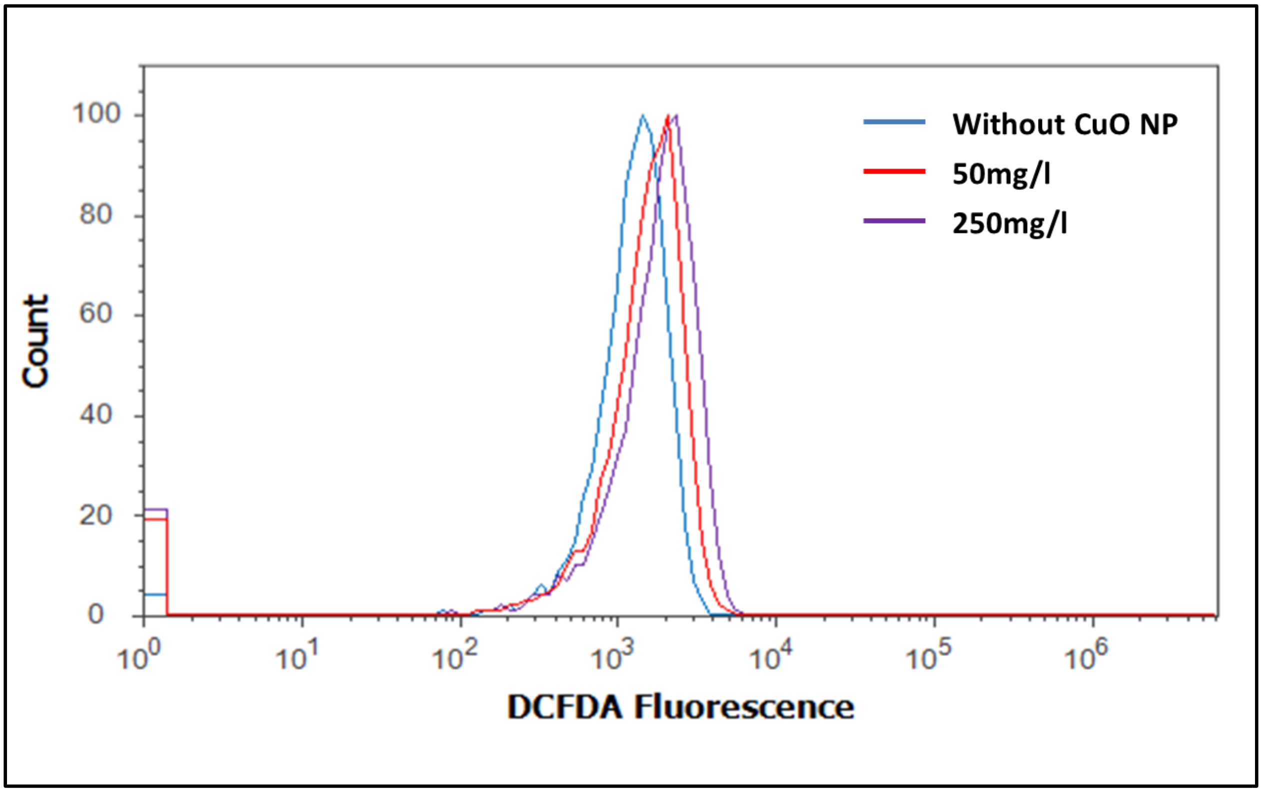

Figure S6: Determination of DCFDA fluorescence level of suspension exposed to different concentration of CuO NP .

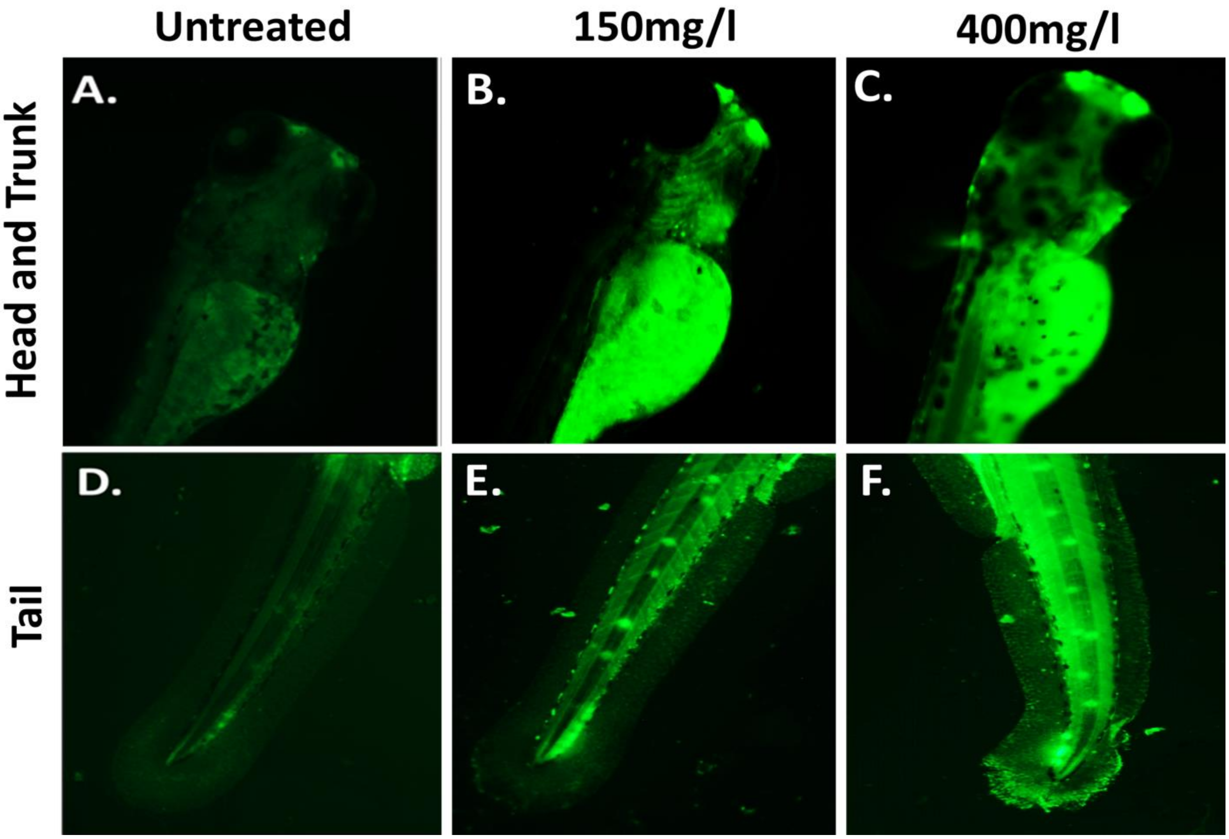

Figure S7: Apoptosis of zebrafish larva cells (96hpf) exposed to different concentration of commercial CuO NP as determined by Acridine orange (AO) staining.

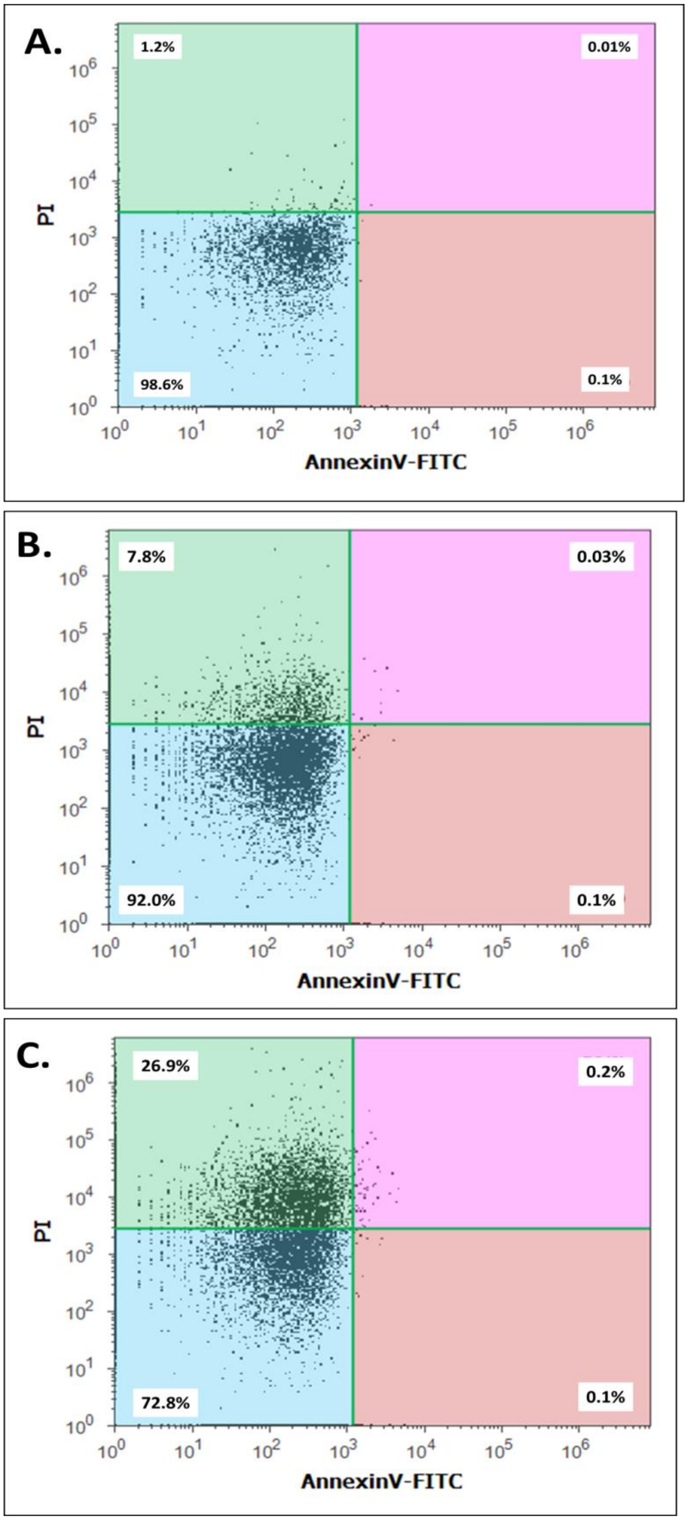

Figure S8: Apoptosis of Zebrafish larva cells (96hpf) exposed to different concentration of commercial CuO NP as determined by flow cytometry analysis by AnnexinV-FITC/PI assay.(A) Untreated (B) 150mg/l exposed embryos (C) 400mg/l exposed embryos.
